# Can Organic Amendments Override Environmental Filtering of Soil Microbiomes? Evidence From 20 Years Old Agricultural Field Trials in Contrasting Tropical Sites

**DOI:** 10.1111/gcb.71061

**Published:** 2026-08-29

**Authors:** Anna M. Visscher, Samuel Mathu Ndungu, Rafaela Feola Conz, Moritz Laub, Monicah Wanjiku Mucheru‐Muna, Daniel Mugendi, Rebecca Yegon, Frank Rasche, Wycliffe Waswa, Mirjam Pulleman, Bernard Vanlauwe, Johan Six, Martin Hartmann

**Affiliations:** ^1^ Soil Biology Group Wageningen University Wageningen the Netherlands; ^2^ Department of Ecology, Radboud Institute for Biological and Environmental Sciences (RIBES) Radboud University Nijmegen the Netherlands; ^3^ International Institute of Tropical Agriculture (IITA) Nairobi Kenya; ^4^ Sustainable Agroecosystems, Department of Environmental Systems Science ETH Zurich Zurich Switzerland; ^5^ Department of Environmental Sciences and Education Kenyatta University Nairobi Kenya; ^6^ Department of Water and Agricultural Resource Management University of Embu Embu Kenya; ^7^ International Center for Tropical Agriculture (CIAT) Cali Colombia

**Keywords:** farmyard manure, integrated soil fertility management, long‐term experiments, nitrogen cycling genes, prokaryotic and fungal communities, sub‐Saharan Africa

## Abstract

Soil microbial communities underpin ecosystem functions critical for sustainable agriculture, yet our understanding of how long‐term management of tropical agroecosystems shapes these communities remains limited. This is particularly the case in sub‐Saharan Africa where soil health challenges are most acute for food security. Using four long‐term (~20 years) experiments across contrasting agroecological zones in Kenya, we studied how organic inputs (farmyard manure, *Tithonia diversifolia* , *Zea mays* stover; applied at 4 Mg C ha^−1^ year^−1^) and nitrogen fertilizer (±120 kg N ha^−1^ per season as calcium ammonium nitrate) affect soil microbial communities. We combined amplicon sequencing of prokaryotic (16S rRNA) and fungal (ITS2) communities with quantification of nitrogen‐cycling functional genes to examine microbial diversity, community composition, and functional potential. Site‐level edaphic properties were the main correlate of community variation, namely 30% and 28% of prokaryotic and fungal β‐diversity, respectively. Despite this strong environmental control, farmyard manure created distinguishable community patterns across all sites, significantly affecting 65 prokaryotic genera and achieving 96% reclassification success for fungal communities. Prokaryotic and fungal communities exhibited contrasting response patterns: prokaryotes responded predominantly to farmyard manure through enrichment of copiotrophic Bacillota (formerly Firmicutes), while fungi were sensitive to both farmyard manure and *T. diversifolia* green manure, recruiting distinct decomposer guilds based on substrate biochemistry. Functional gene responses were amplified at the driest, most nutrient‐poor site, where farmyard manure led to a 30‐fold increase in the abundance of ammonia‐oxidizing bacteria compared to the control treatment. Mineral nitrogen fertilization alone did not produce distinct community composition but modestly reduced specific nitrogen‐cycling genes. This demonstrates that organic resource management, not mineral inputs, drives long‐term microbial community development. These findings provide decadal‐scale evidence that sustained organic amendments can generate predictable microbial responses across environmentally heterogeneous tropical landscapes, informing integrated soil fertility management strategies for sub‐Saharan Africa.

## Graphical abstract

Across four long‐term Kenyan maize trials, site was the dominant driver of microbial community composition and N‐cycling gene abundance. Among organic amendments, farmyard manure most strongly enriched copiotrophic taxa and raised prokaryotic diversity. Mineral nitrogen had limited effects; functional gene responses were strongest at the driest, most nutrient‐poor site.

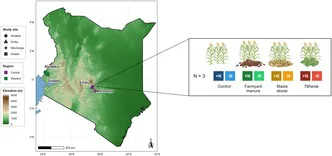

## 1 Introduction

Soil degradation in sub‐Saharan Africa represents a convergence of multiple, mutually reinforcing threats to agricultural sustainability, including nutrient depletion from continuous cropping without adequate replenishment (Kihara et al. 2020; Vanlauwe et al. 2015), erosion exacerbated by intense rainfall patterns, faster organic matter decomposition under warm climatic conditions, and increasing vulnerability to climate variability (Couëdel et al. 2025; Cherubin et al. 2025). These compounding conditions create what has been termed a “perfect storm” for soil health degradation in the region (Tittonell and Giller 2013). Millions of smallholder farmers in sub‐Saharan Africa are disproportionally affected by soil degradation and fertility decline, while meeting local food security and the growing global demand for tropical commodity products is putting further pressure on already fragile crop lands (Nguyen et al. 2023). Yet, smallholder farmers in low‐and middle‐income countries are largely ignored when it comes to investment in agricultural research for development. Together these impacts cascade to food, income and water insecurity, deepening existing social inequalities. Climate change further exacerbates degradation, while degraded soils increase vulnerability to climate (Ortiz‐Bobea et al. 2021; Couëdel et al. 2025).

Soil health, defined as the continued capacity of soil to function as a vital living ecosystem that sustains plants, animals, and humans (Doran and Zeiss 2000), encompasses physical, chemical, and biological dimensions (Creamer et al. 2022). While biological indicators, particularly microbial community structure and function, are increasingly recognized as sensitive, integrative measures of soil health status (Vazquez et al. 2025; Bünemann et al. 2018; Karlen et al. 2019), a pronounced geographic bias exists in this knowledge base (Cherubin et al. 2025). The vast majority of research on soil biological indicators has been conducted in temperate agroecosystems of the Global North (Lehmann et al. 2020; Barrios 2007). The ecological paradigms derived from these temperate agroecosystems, where soils have high organic matter content and strong buffering capacity, often fail to predict microbial dynamics in tropical systems. In heavily weathered, nutrient‐poor tropical agroecosystems, the interplay between rapid thermal decomposition and severe nutrient depletion can lead to highly divergent, context‐dependent microbial responses to agricultural management. This leaves critical blind spots in our understanding of how these indicators respond to management in tropical agroecosystems, particularly in resource‐constrained smallholder farming systems across sub‐Saharan Africa where soil health challenges are most acute (Cowan et al. 2022).

Soil microbial communities exhibit strong biogeographic patterns controlled primarily by edaphic factors such as pH, texture, and organic matter content, alongside climatic variables (Delgado‐Baquerizo et al. 2018; Fierer et al. 2012; Fierer and Jackson 2006). though bacteria and fungi may respond differently to environmental versus management drivers (Barreiro et al. 2022; Fox et al. 2021). These environmental factors act as abiotic filters that fundamentally structure which microbial taxa can establish themselves and persist in a given soil environment (Martiny et al. 2006). Within these edaphic constraints, agricultural management practices, particularly organic carbon and nutrient amendment strategies, act as secondary filters that further shape microbial community composition and functional potential (Banerjee et al. 2019; Hartmann et al. 2015). Agriculturally relevant microbes from both bacterial and fungal domains drive critical soil processes, including nutrient cycling, decomposition, nutrient uptake, and plant stress tolerance (Hartmann and Six 2023; Levy‐Booth et al. 2014; Reed et al. 2011; Van Der Heijden et al. 2008). Recurrent microbial responses to organic and mineral amendments have been documented, including shifts toward copiotrophic taxa under labile carbon inputs and changes in key functional groups, such as increased nitrifiers under mineral fertilization and enhanced decomposers under organic amendments (Francioli et al. 2016; Ding et al. 2016). Whether these taxonomic responses translate to functional differences, or whether functional redundancy maintains similar capacities across distinct communities, remains unclear (Louca et al. 2018). This raises a critical question regarding the hierarchical relationship between site and management effects: do site‐specific edaphic and climatic contexts determine the overall microbial potential, with management practices modulating the extent to which this potential is realized? Alternatively, can management practices override site constraints in shaping microbial communities and functions in similar ways, despite variation in local environmental contexts? The latter scenario would facilitate the formulation of soil health management recommendations for smallholder farmers, while the former would necessitate context‐specific approaches tailored to each agroecological setting. Despite the importance of long‐term experiments for distinguishing transient from persistent management effects (Hartmann and Six 2023), such data remain scarce in sub‐Saharan Africa, leaving these fundamental questions unresolved regarding the universality versus context‐dependency of microbial responses to soil fertility management.

Strategies such as “Integrated Soil Fertility Management” (ISFM) and “sustainable intensification” seek to balance productivity with sustainability by combining mineral fertilizers with organic resource inputs (Pretty et al. 2012; Vanlauwe et al. 2010). This dual‐input approach leverages complementary benefits, including readily available nutrients from mineral sources and sustained nutrient release, improved soil structure, and enhanced biological activity from organic amendments (Chivenge et al. 2009; Bulluck et al. 2002; Palm et al. 2001). However, the quality of organic resources, defined by their C:N ratio, lignin content, and polyphenol composition, fundamentally determines their effects on soil properties and microbial responses (Manzoni et al. 2012; Palm et al. 2001). Readily decomposable materials such as farmyard manure (FYM) represent a special case: beyond their low C:N ratio that favors rapid decomposition, manure introduces substantial microbial inocula directly into the soil, including copiotrophic populations already adapted to nutrient‐rich conditions and primed for immediate activity, as well as methanogens that may influence soil carbon stabilization (Liu et al. 2020; Ho et al. 2017; Fierer et al. 2007). In contrast, more recalcitrant plant‐based inputs like maize stover may favor oligotrophic taxa with slower growth strategies and greater efficiency under nutrient limitation (Fanin et al. 2013; Pascault et al. 2013). Green manures such as *Tithonia diversifolia* represent another fast‐decomposing input, with high N and P concentrations that promote rapid nutrient release (Jama et al. 2000). However, unlike FYM, plant‐based green manures do not introduce substantial exogenous microbial populations, meaning their effects on soil communities operate primarily through substrate provision rather than direct inoculation.

The global inconsistency of these microbial responses to fertilizer management becomes evident when comparing long‐term agricultural trials across different climate zones. In well‐buffered temperate systems, such as the DOK trial in Switzerland (Mäder et al. 2002), decades of organic and mineral fertilization have yielded highly structured, predictable trajectories in soil biological quality, where organic inputs such as farmyard manure consistently increased microbial biomass (Krause et al. 2022) and distinctively shifted community structures compared to exclusive mineral fertilization (Hartmann et al. 2015). Conversely, case studies from sub‐Saharan Africa demonstrate highly variable microbial community responses to organic amendments across sites (Köberl et al. 2022; Diallo‐Diagne et al. 2016; Partey et al. 2013), while mineral N fertilization shows more consistent effects than organic amendments, these include long‐term soil acidification (Tian and Niu 2015) and shifts in microbial community composition (Geisseler and Scow 2014), with microbial biomass reductions occurring indirectly where acidification drives soil pH below approximately 5. Long‐term trials in Kenya examining organic and mineral amendment strategies for 20 years have revealed that combining farmyard manure with mineral nitrogen maintains maize yields and partially slows soil organic carbon decline, though even the best‐performing treatments, adding very high amounts of carbon (4 Mg C ha^−1^ year^−1^) through external organic inputs, have proven insufficient to maintain initial carbon stocks at most sites, with relatively low carbon storage efficiencies (i.e., below 20%) (Laub, Corbeels, Ndungu, et al. 2023; Laub, Corbeels, Couëdel, et al. 2023). These findings on long‐term agronomic and carbon storage outcomes underscore the need to examine the biological dimensions of mediating these soil processes. Beyond these agronomic and carbon storage outcomes, site‐specific studies within these trials have shown that soil texture modulates ammonia‐oxidizing prokaryote responses to organic input quality, with *amoA* gene dynamics of AOB and AOA differing between the clayey and sandy sites (Muema et al. 2015; Muema, Cadisch, and Rasche 2016; Muema, Cadisch, Musyoki, et al. 2016). However, a comprehensive assessment across all four sites integrating full microbial community profiling with nitrogen‐cycling functional potential remains lacking.

Using four long‐term trials (approximately 20 years) across contrasting agroecological zones in Kenya, we investigated how organic resource amendments and mineral nitrogen fertilization shape soil microbial community structure and functionality. Furthermore, we tested whether these microbial responses are universal across the contrasting soil textures and climatic contexts, or whether site‐specific agroecological conditions override treatment effects. By coupling microbial community profiling (16S rRNA gene and ITS2 rrn amplicon sequencing) with quantification of key nitrogen cycling genes, we assessed how two decades of differential organic matter and mineral nitrogen management have affected both taxonomic diversity and functional potential of soil microbial communities. Based on the distinct biochemical properties of the inputs and soil textural variations across the sites, we hypothesized that (1) the quality of organic amendments determines the taxonomic and functional response of soil microbiome, but this management effect is fundamentally modulated by site‐specific soil texture. Specifically, due to its low C:N ratio and direct microbial inoculation, farmyard manure will induce a taxonomically broad, highly distinct restructuring of the soil microbiota across all sites, while stimulating nitrogen cycling guilds primarily in coarse‐textured, poorly buffered sandy soils where substrate limitation is more pronounced. Conversely, plant‐based materials will trigger narrower, substrate‐specific responses. Conversely, plant‐based materials will trigger narrower, substrate‐specific responses. The high‐nitrogen, low C:N green manure Tithonia will favor fast‐growing copiotrophic taxa, whereas the nitrogen‐poor, high C:N maize stover will primarily stimulate fungal decomposers and enhance overall microbial abundance in fine‐textured clayey soils where organic matter is stabilized.

We further hypothesized that (2) fungal and prokaryotic communities will display contrasting patterns of structural sensitivity and taxonomic breadth following two decades of management. Due to their specialized enzymatic capacity to degrade complex plant polymers, fungal communities will show more pronounced structural discrimination between distinct inputs than prokaryotes. However, prokaryotes will display a taxonomically more widespread response across different phyla, driven by their metabolic versatility and the direct, long‐term introduction of exogenous taxa via manure.

## 2 Materials and Methods

### 2.1 Study Site Description

**FIGURE 1:**
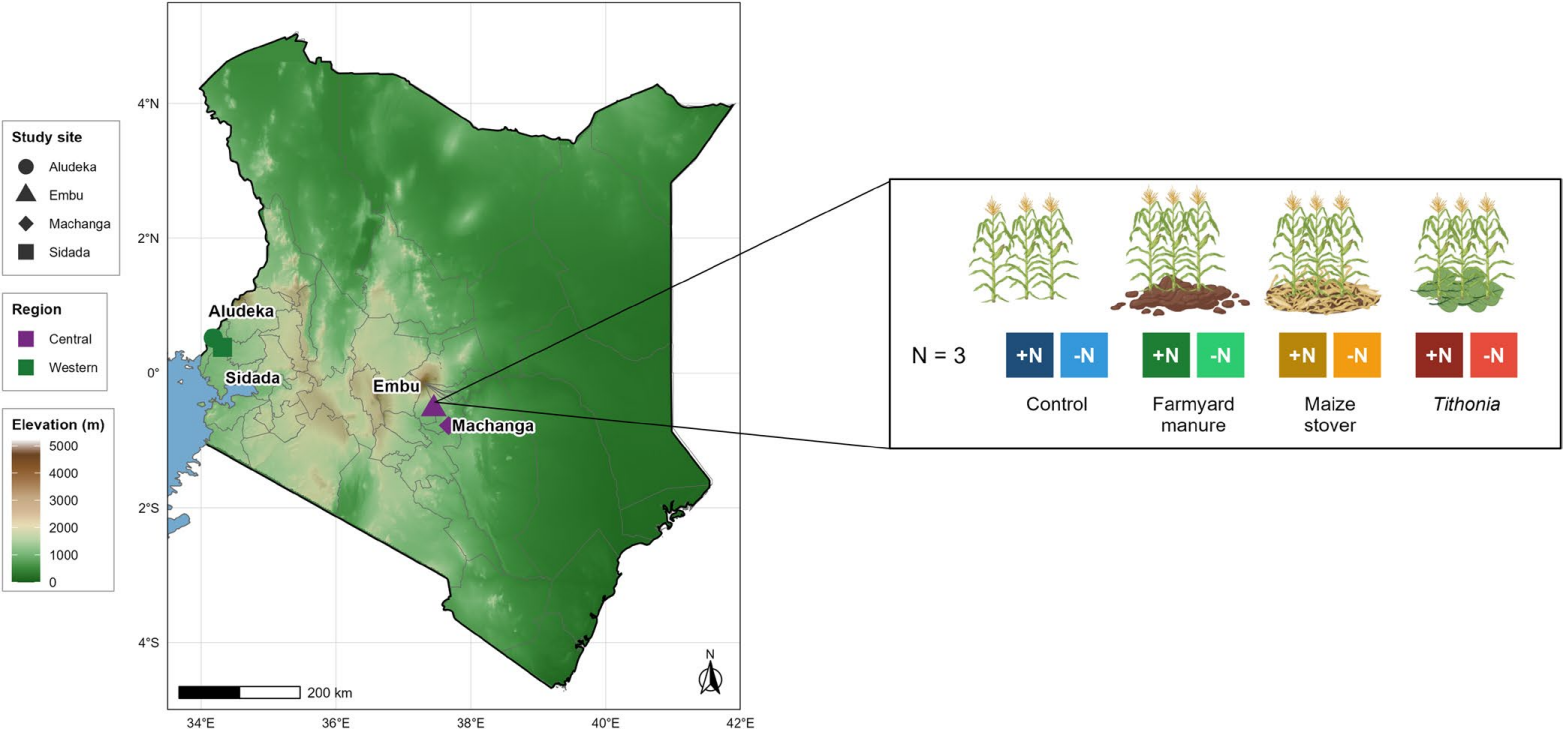
Visual representation of the experimental trials in central and western Kenya, which aimed to evaluate the effects of organic inputs and mineral N application on functional genes and microbial communities across different agroecological zones. The digital elevation model (DEM) indicates the location of the four long‐term trials, with coloring indicating elevation from green (low) to dark brown (high). At each site, monoculture *Zea mays* was either amended with farmyard manure (green), *Z. mays* stover (yellow), or *Tithonia diversifolia* (red), or not amended (blue). Each treatment received either mineral N application (darker color) or not (lighter color). The schematic represents a simplified layout; treatments were randomized within each block.

This study examined four long‐term experiments conducted in central and western Kenya (Figure 1). The experiments in central Kenya (Embu and Machanga) were established in 2002 and those in western Kenya (Sidada and Aludeka) in 2005 (Laub, Corbeels, Ndungu, et al. 2023; Laub, Corbeels, Couëdel, et al. 2023). All sites have been continuously cropped with maize, with two growing seasons per year: a long rainy season (March to August/September) and a short rainy season (September/October to January/February). The four sites were selected to represent varying altitudes, precipitation, temperatures, and soil conditions. Sidada (1420 m.a.s.l., 1730 mm, 22.6°C) and Aludeka (1180 m.a.s.l., 1660 mm, 24.4°C) have the longest rainy seasons and highest annual rainfall, providing favorable conditions for maize production. Embu (1380 m.a.s.l., 1175 mm, 20.1°C) and Machanga (1022 m.a.s.l., 795 mm, 23.7°C) are drier, with maize production in Machanga experiencing a high risk of crop failure. All sites are characterized by heavily weathered soils, with low pH (5.3 to 5.5). The soils at Embu (Humic Nitisol) and Sidada (Humic Ferralsol) both have high clay content, while Aludeka (Haplic Acrisol) and Machanga (Ferric Alisol) are coarse textured (Table 1). The landscapes are mostly flat with gentle slopes (1°–5°), but the plots at Embu are terraced to create a flat surface. Land‐use history varied across sites. Sidada and Aludeka were previously savanna ecosystems with about 50% tree cover, and were converted to low intensity, shifting cultivation 25 years before the start of the experiments in 2005. Embu was originally a tropical evergreen forest cleared 50–100 years ago for shifting cultivation. Machanga was a steppe grassland with 20%–30% tree cover and was subject to intensive cattle grazing prior to trial establishment in 2002.

**TABLE 1:** Location, soil properties and climatic conditions of four study sites in Kenya.

Soil characteristics	Unit	Embu	Machanga	Sidada	Aludeka
Latitude	Decimal degrees	0.517° S	0.793° S	0.143° N	0.574° N
Longitude	Decimal degrees	37.459° E	37.664° E	34.422° E	34.191° E
Altitude	m.a.s.l.	1380	1022	1420	1180
Initial soil C	g kg^−1^	29	3	15	8
Initial total N	g kg^−1^	3	0.2	1.2	0.8
Bulk density	g cm^−3^	1.26	1.51	1.30	1.45
PH‐H_2_O	—	5.43	5.27	5.4	5.49
Clay	g kg^−1^	598	132	557	134
Soil type	IUSS working group, 2024	Humic Nitisol	Ferric Alisol	Humic Ferralsol	Haplic Acrisol
Slope	%	5	2.5	2	1
Mean annual rainfall	mm	1175	795	1730	1660
Mean annual temperature	°C	20.1	23.7	22.6	24.4

*Note:* Soil properties are provided for the 0–15 cm depth layer and are based on measurements prior to the start of the experiment in early 2002 for Embu and Machanga, and early 2005 for Aludeka and Sidada. Coordinates are given in the WGS 84 reference system.

*Source:* Adapted from Laub, Corbeels, Couëdel, et al. (2023) under the CC BY license (http://creativecommons.org/licenses/by/4.0/).

### 2.2 Experimental Set‐Up and Management

All four long‐term trials used an identical split‐plot design with three replicated blocks (Figure 1). The main plot factor was organic inputs, and the split‐plot factor was mineral N application. The study involved the application of different organic resources at rates of 4 Mg C ha^−1^ year^−1^. While a lower input intensity of 1.2 Mg C ha^−1^ year^−1^ also exist within the long‐term trial design, this treatment was excluded from the present study due to resource constraints and a deliberate sampling strategy to target the highest amendment rate, thereby maximizing the capacity to detect distinct, persistent treatment signatures and establish an upper boundary for management legacies after two decades of sustained inputs (Laub, Corbeels, Ndungu, et al. 2023). The sub‐plot treatment compared the addition 120 kg N ha^−1^ per season of mineral N in the form of calcium ammonium nitrate (nitrochalk) with no mineral N application. The organic resources included farmyard manure from cows (FYM), leaves and small stems (< 2 cm diameter) of Tithonia (*T. diversifolia*, TD), and maize stover ( *Zea mays* , MS) as well as a control without any organic residue input (Table S2). They were selected to represent a broad range of organic resource qualities, defined by C‐to‐N ratio (C/N), lignin content, and polyphenol content (Palm et al. 2001). Organic resources were applied annually to plots (6 × 5 m per split‐plot in Embu; 6 × 6 m at all other sites) before maize sowing in the long rainy season and incorporated into the soil to a depth of approximately 15 cm using hand hoes. For further details on the categorization of the organic resources used, we refer to Chivenge et al. (2009) and Laub, Corbeels, Ndungu, et al. (2023). Before each season, all plots received a uniform application of 60 kg P ha^−1^ and 60 kg K ha^−1^ at planting to ensure that P and K were not limiting (Laub, Corbeels, Ndungu, et al. 2023). Additional details regarding plant density, thinning, weeding, and general management practices are available in Laub, Corbeels, Ndungu, et al. (2023).

### 2.3 Soil Sampling

Soil sampling from the four experimental sites was conducted in 2021 as described by Laub, Corbeels, Ndungu, et al. (2023). Briefly, soil samples were collected before planting of the 2021 long rainy season for Aludeka, Sidada, and Machanga, and after the harvest of the 2021 long rainy season for Embu. To minimize disturbance to the experimental plots, samples were collected using a single soil core from the center of the cropped area in each plot. At Embu, a gauge auger of 60 mm diameter and 500 mm length was used (Eijkelkamp Soil & Water, Giesbeek, the Netherlands), while at the other sites, a soil corer of 55 mm diameter and 1000 mm length was employed (Giddings Machine Company, Windsor, USA). Different equipment was necessary at Embu due to the specific soil conditions (Laub, Corbeels, Ndungu, et al. 2023; Laub, Corbeels, Couëdel, et al. 2023). All sites were sampled at a comparable pre‐planting stage, three before the 2021 long rains and Embu before the short rains, where hard, dry pre‐sowing soil required sampling at the end of the preceding season once amendments had decomposed and maize residues were removed.

Per plot, a composite soil sample was assembled by collecting soil cores across the plot until approximately 1 kg of fresh soil was obtained. The cores were pooled and thoroughly homogenized by hand‐mixing in a clean container, with visible roots and stones removed. A 250 g aliquot was then taken from this homogenized composite sample, placed in an airtight Ziploc bag, stored in cooler boxes with ice packs, and transported to Nairobi for storage at −20°C prior to nitrate, ammonium, and microbial community analyses. A separate 50 g aliquot was taken from the same homogenized composite sample for soil moisture determination by oven drying at 105°C. The remaining soil was air‐dried at room temperature, sieved through a 2 mm mesh, and thoroughly mixed before being packaged in airtight Ziploc bags and stored in a cool, dry place pending further physical and chemical analyses.

### 2.4 Physicochemical Soil Properties

Soil samples were divided for physicochemical analysis. Nitrate (${\mathrm{NO}}_{3}^{-}$) and ammonium (${\mathrm{NH}}_{4}^{+}$) were determined photometrically on KCl extracts at the Mazingira Centre laboratory, International Livestock Research Institute (ILRI), Nairobi, Kenya, following Hood‐Nowotny et al. (2010). All other physicochemical analyses (pH‐H_2_O, TOC, TN, and BD) were conducted as described in Laub, Corbeels, Ndungu, et al. (2023).

### 2.5 Microbial Gene Abundance

Frozen soil samples were shipped under cool conditions to the Sustainable Agroecosystems Laboratory at ETH Zürich, Switzerland. DNA extraction from 0.25 g fresh soil was performed with the DNeasy PowerSoil Pro Kit (Qiagen, Hilden, Germany) following the manufacturer's instructions and using the QIACube system (Qiagen). Samples with higher clay content, such as soils from the Embu site, were pretreated by adding 100 μL of ATL buffer combined with 700 μL of solution CD1 from the DNeasy PowerSoil Pro kit (Qiagen) during the first step of soil aggregate and cell disruption, followed by heating at 65°C for 10 min. The remaining samples were processed according to the manufacturer's protocol using 800 μL of CD1 solution. All samples were placed in a FastPrep‐24 5G instrument (MP Biomedicals, Santa Ana, CA, USA) and lysed at 5 m/s for 30 s twice. After extraction, DNA quantity and quality were measured by UV/Vis spectrophotometry with the QIAxpert system (Qiagen).

The abundance of taxonomic groups (bacteria and archaea, hereafter termed prokaryotes, and fungi) and functional groups involved in nitrogen cycling was assessed using a SYBR Green‐based quantitative PCR (qPCR) approach. Specifically, gene abundances were quantified for total prokaryotes (16S rRNA genes, henceforth 16S), total ammonia oxidizers (archaeal and bacterial amoA genes), nitrogen fixers (*nifH* gene), and denitrifiers (*nirK*, *nirS*, and *nosZI* genes). Potential amplification inhibition induced by unintentional co‐extraction of contaminants was tested across all samples by spiking pGEM‐T plasmid (GenBank Accession No. X65308; Promega, Madison, WI, USA) into the soil DNA at equimolar concentrations and amplifying a region on the plasmid using the vector‐specific primers SP6 and T7 (Microsynth, Balgach, Switzerland). To check for inhibition, the spiked plasmid was simultaneously amplified in pure water controls. Quantification cycle (*Cq*) values revealed no significant differences in mean (ANOVA, *p* = 0.734) or variance (Levene, *p* = 0.114) between the water baseline and soil DNA extracts. Real‐time amplification curves confirmed identical kinetic efficiencies, showing parallel exponential slopes and overlapping Cq cycles (Figure S1). Individual sample variations remained tightly clustered around the baseline mean (20.17 ± 0.33 SD), with 100% of soil extracts falling within ±1.0 *Cq* cycle. This demonstrates the absence of both systemic and sample‐specific PCR inhibition across the extracted soil matrices.

The qPCR standards were produced from purified amplicons of each target gene obtained by pooling normalized DNA from all samples. For the standard curves, concentrations ranging from 10^−3^ to 10^−7^ ng of target template were utilized for all reactions. Primers targeting taxonomic marker genes (16S rRNA gene V4 region) and functional genes involved in nitrogen cycling (archaeal and bacterial ammonia oxidation, nitrogen fixation, denitrification, and nitrous oxide reduction) are detailed in Table S1.

All qPCRs were performed in a final volume of 20 μL containing 0.4 μM of each primer, 1× SsoAdvanced Universal SYBR Green Supermix (Bio‐Rad Laboratories, Hercules, CA, USA), and 20 ng of template DNA. The cycling conditions for all reactions consisted of a polymerase activation step at 98°C for 3 min, followed by denaturation at 95°C for 15 s, annealing at primer‐specific temperatures (Table S1) for 30 s, and elongation at 72°C for 15 s, repeated for the number of cycles specified in Table 1. To verify amplification specificity, melting curves were generated at the end of the amplification cycles.

All qPCRs were performed in technical triplicates using a CFX96 Touch Real‐Time System thermocycler (Bio‐Rad Laboratories, Hercules, CA, USA), and the results were documented and analyzed using CFX Maestro software (Bio‐Rad Laboratories, Hercules, CA, USA). To check for between‐run differences, standard samples were repeated in every plate and compared. The qPCR efficiencies were between 65% and 98% with an *R* ^2^ ≧ 0.99 (Figure S2 and Table S3), the lowest being for *nirK*, whose abundances are therefore interpreted with corresponding caution.

The estimated copy numbers for each target gene were calculated using the following formula as described by Longepierre et al. (2021):

$$\text{Number of copied}\;\mathrm{per}\;\mathrm{\mu L}=\frac{6.022\times 10^{23}\left(\text{molecules}/\text{mole}\right)\times \mathrm{DNA}\;\text{concentrations}\left(g/\mathrm{\mu L}\right)}{\text{Number of base pairs of the target gene}\times 660\;\left(\text{Daltons}\right)}$$

where 6.022 × 10^23^ is Avogadro's number and 660 is the average weight of a single base pair.

### 2.6 Microbial Community Structure

Changes in prokaryotic and fungal community structures were investigated using DNA metabarcoding of ribosomal markers. PCR amplification of the prokaryotic 16S rRNA gene (V3‐V4 region) and fungal ITS2 region of the rrn operon were performed using primers detailed in Table S1. All primers included the sequencing primer sites of the Nextera Illumina adapters.

PCRs contained 1× GoTaq G2 Hot Start Colorless Master Mix (Promega, Madison, WI, USA), 2.5 to 3.0 mM MgCl_2_, 0.4 μM of each primer, and 40 ng of DNA template in a final volume of 25 μL. Cycling conditions for the PCRs consisted of a polymerase activation step at 95°C for 5 min, followed by 30 cycles (16S) or 35 cycles (ITS) of denaturation at 95°C for 40 s, primer hybridization at 58°C (16S) or 55°C (ITS) for 40 s, and elongation at 72°C for 1 min, with a final elongation at 72°C for 10 min. PCR amplification was carried out in technical triplicates, and triplicates were pooled prior to sequencing. Amplification quality was analyzed via capillary electrophoresis with the QIAxcel Advanced system (Qiagen).

The pooled amplicons were sent to the Functional Genomics Center Zurich (FGCZ, Zurich, Switzerland) for indexing PCR. Indexed amplicons were purified, quantified, and pooled in equimolar ratios. Pre‐sequencing on the Illumina MiniSeq platform (Illumina Inc., San Diego, CA, USA) was performed to inform library re‐pooling in order to achieve optimal read count distributions across all samples. Final sequencing was executed on the Illumina NextSeq 2000 platform (Illumina Inc.) using paired‐end 300 bp reads. The raw sequences were deposited in the European Nucleotide Archive (ENA) under the accession number PRJEB101818 (https://www.ebi.ac.uk/ena/browser/view/PRJEB103795).

### 2.7 Bioinformatics

Sequencing data were processed using a customized bioinformatics pipeline largely based on VSEARCH v2.21.1 (Rognes et al. 2016) as published previously (Longepierre et al. 2021). In brief, PCR primers were trimmed with CUTADAPT v3.4 (Martin 2011), allowing for one mismatch. Bowtie2 v2.4.5 (Langmead and Salzberg 2012) was used to filter for PhiX contamination by aligning the reads against the PhiX genome (accession NC_001422.1). The *fastq_mergepairs* function in VSEARCH was used to merge trimmed paired‐end reads. The *fastq_filter* function in VSEARCH was applied for quality filtering with a maximum expected error of 1 (Edgar and Flyvbjerg 2015). Sequences were dereplicated using the *derep_fulllength* function in VSEARCH and delineated into amplicon sequence variants (ASVs) by applying the UNOISE algorithm (Edgar 2016) of VSEARCH with an alpha of 2 and a minsize of 8. The UCHIME3 algorithm (Edgar 2016) implemented as the *uchime3_denovo* function in VSEARCH was used to identify and remove potentially chimeric ASV sequences with an abundance skew of 8. Remaining ASV sequences were tested for the presence of ribosomal signatures using Metaxa2 v2.2.3 (Bengtsson‐Palme et al. 2015) for the 16S rRNA gene and ITSx v1.1.3 (Bengtsson‐Palme et al. 2013) for the ITS2 sequences. The final ASV table was generated by mapping the quality‐filtered reads of each sample against the verified ASV sequences using the *usearch_global* algorithm implemented in VSEARCH with the settings maxhits 1, maxrejects 100, maxaccepts 0, and a minimum identity of 97%.

Verified ASV sequences were taxonomically classified using the SINTAX algorithm (Edgar 2016) of VSEARCH against the SILVA v138.2 database (Quast et al. 2013) for 16S rRNA gene sequences and against the UNITE v10.0 database (Abarenkov et al. 2010; Nilsson et al. 2019) for ITS2 sequences, using a bootstrap cutoff of 0.8. Both reference databases were trimmed to the amplicon regions using an in silico PCR approach with CUTADAPT prior to classification to improve accuracy. ASVs not assigned at the domain level of bacteria, archaea, or fungi, as well as ASVs assigned to organelle structures (chloroplasts and mitochondria), were removed from the ASV table.

### 2.8 Statistical Analyses

All statistical analyses were performed in R version 4.1.3 (R Core Team 2022) using RStudio version 2022.02.1+461 (RStudio Team 2022). For all tests, a *p*‐value < 0.05 was considered statistically significant unless otherwise stated. Sequencing depth was assessed using rarefaction curves with the *rarecurve* function in the vegan package v.2.6‐2 (Oksanen et al. 2022). To account for variations in sequencing depth, quality‐filtered ASV count tables were normalized using an iterative subsampling approach (Schloss 2024). Specifically, samples were rarefied to the minimum sequencing depth 100 times, and the resulting subsampled ASV tables were averaged into a single normalized table to minimize stochastic noise and prevent the loss of data robustness associated with a single rarefaction step (Martiny et al. 2017; Hemkemeyer et al. 2019). All α‐diversity indices and β‐diversity dissimilarity matrices were then calculated from this averaged table, as a robustness check, these metrics were also computed from the individual subsampled matrices, which gave consistent results, with only fungal richness showing minor sensitivity. A fixed random number seed was set to ensure reproducibility of the rarefaction procedure. All statistical visualizations were created using ggplot2 v.3.3.6 (Wickham 2016) with ggtext v.0.1.1 (Wilke 2020) for styled text labels and patchwork v.1.1.1 (Pedersen 2020) for multi‐panel figure composition.

The effects of organic resource management (FYM, MS, TD, CT), mineral N input (+N, −N), site (Aludeka, Embu, Machanga, Sidada), and their interactions on α‐diversity, β‐diversity, and functional gene abundances (16S, archaeal and bacterial *amoA*, *nifH*, *nirK*, *nirS*, *nosZI*) were assessed using permutational analysis of variance (PERMANOVA, Anderson 2001). For α‐diversity, Euclidean distance matrices were used with α‐diversity metrics including observed richness (calculated using the *specnumber* function), Shannon diversity (using the *diversity* function), and Pielou's evenness (Shannon diversity divided by log of observed richness), all implemented in *vegan*. For β‐diversity, Bray‐Curtis dissimilarities were calculated from the normalized ASV count tables via the *vegdist* function in *vegan* to assess changes in community composition.

For all soil parameters, functional genes and diversity analyses, the full factorial model included all main effects and interactions (~Treatment (FYM, MS, TD, CT) × Ninput (+N, −N) × Site (Aludeka, Embu, Sidada, Machanga)) with replicate block as strata, using 999 permutations as implemented in the *adonis2* function of *vegan*. Homogeneity of multivariate dispersions was tested using permutational analysis of multivariate dispersions (PERMDISP, Anderson 2006) via the *betadisper* and *permutest* functions in *vegan* with 999 permutations, applied to all main effects and interactions. Pairwise comparisons between organic resource management were performed using PERMANOVA (*adonis2* function, *vegan* package) applied to all treatment pairs with 999 permutations, blocked by replicate, and *p*‐values were used to generate compact letter displays with the *multcompLetters* function from the *multcompView* package v.1.4‐18 (Hothorn et al. 2022). Overall analyses included all sites combined, and site‐specific analyses were performed separately for each site using the model ~ Org Res*Treatment* × *N*input with replicate block as strata.

Differences in β‐diversity were visualized using canonical analysis of principal coordinates (CAP) (Anderson and Willis 2003) implemented as the *CAPdiscrim* function in the BiodiversityR package v.2.14‐1 (Kindt 2022). CAP analysis was constrained by organic resource treatment only. The optimal number of principal coordinate axes (*m*) was selected to maximize classification success between treatment groups while avoiding overfitting that could result from including excessive axes, with models evaluated for statistical significance (*p* < 0.05). Statistical significance of CAP models was assessed using permutation tests (999 permutations), and the strength of treatment discrimination was quantified using Pillai's trace. The CAP reclassification success rate provided a quantitative estimation of the degree of discrimination between treatment groups. Separate CAP analyses were performed for prokaryotes (16S) and fungi (ITS).

To identify microbial taxa responding strongly to organic resource management, ASVs were aggregated to the genus level by summing read counts of ASVs assigned to the same taxonomic group. For each genus, PERMANOVA (*adonis2* function, *vegan* package) was used to test the effect of organic resource treatment on relative abundance, with Euclidean distance matrices calculated from genus‐level relative abundances and 999 permutations. To adjust for multiple testing, *p*‐values were corrected using the Benjamini‐Hochberg false discovery rate (FDR) method (Benjamini and Hochberg 1995) via the *p*.adjust function in R. Genera with *F*‐ratio ≥ 10 and FDR‐adjusted *p* < 0.05 were considered significantly responsive to organic resource treatment and included in visualization. The *F*‐ratio threshold was explicitly utilized as a rigorous effect‐size filter. Because the *F*‐statistic reflects the ratio of variance explained by the organic management treatments relative to the residual variation, this stringent baseline selectively prunes the dataset to focus exclusively on highly responsive, dominant microbial drivers while filtering out minor statistical fluctuations. No arbitrary maximum threshold was applied. Pairwise comparisons between each organic resource treatment (FYM, MS, TD) and the control (CT) without organic resource amendment were performed separately for each selected genus by iteratively applying PERMANOVA (*adonis2* function, vegan package) to each treatment pair with Euclidean distance matrices and 999 permutations. Genera were considered significantly different from the control at *p* < 0.05 for each pairwise comparison. For visualization, treatment effects were expressed as log2 fold‐change in genus relative abundance relative to the unamended control, using a pseudocount of half the smallest non‐zero relative abundance to accommodate genera absent from control plots. A value of zero therefore indicates no difference from the control. Potential lifestyle traits of the identified taxa were inferred through literature review, supported by annotations from FAPROTAX v1.2.4 (Louca et al. 2016) and FUNGuild v1.0 (Nguyen et al. 2016) for prokaryotes and fungi, respectively.

The effects of soil physicochemical properties (${\mathrm{NH}}_{4}^{+}$, ${\mathrm{NO}}_{3}^{-}$, bulk density, total organic carbon, total nitrogen, pH) and functional gene abundances (16S, archaeal and bacterial *amoA, nifH, nirK, nirS, nosZI*) on prokaryotic and fungal community composition were assessed using distance‐based redundancy analysis (db‐RDA) (Legendre and Anderson 1999) via the *capscale* function in vegan based on Bray‐Curtis dissimilarities. Parsimonious models were constructed using forward stepwise selection with the *ordistep* function in vegan, starting from a null model (community ~1) and building toward a full model including all valid environmental variables. Model significance was tested using PERMANOVA with 999 permutations, and adjusted *R* ^2^ values were calculated. Overall analyses included all sites combined, and site‐specific analyses were performed separately for each site, retaining only environmental variables with non‐zero variance at each site.

## 3 Results

### 3.1 N‐Cycling Functional Gene Abundance

Site was the dominant driver of variation in functional gene abundances. Site explained 36%–62% of variance across all measured genes, with the strongest site controls over *nirK*, *nifH*, and 16S (Table 2). Organic amendment significantly influenced six of the seven measured functional genes (all except *nifH*), though organic resource treatment effect sizes remained substantially smaller than site effects (5%–14% of variance). Org Res*Treatment* by *Site* interactions were pronounced (7%–12% of variance) and occurred for six genes (16S, archaeal and bacterial *amoA*, *nifH, nirK, nosZI*), indicating that organic amendment effects varied considerably by site. Heterogeneous dispersion in site effects for archaeal and bacterial *amoA*, and *nosZI* warrant cautious interpretation of site‐level patterns, as differences may partially reflect variance heterogeneity rather than solely site effects (Table 2).

**TABLE 2:** Effects of organic resource treatment, mineral N application, site, and their interactions on soil chemical properties and functional gene abundances assessed by permutational analysis of variance (PERMANOVA) and permutational analysis of multivariate dispersion (PERMDISP).

		OR treatment (T)			Mineral N application (N)			Site (S)			T × S		
		*R* ^2^	P	D	*R* ^2^	P	D	*R* ^2^	P	D	*R* ^2^	P	D
${\mathrm{NH}}_{4}^{+}$	mg kg^−1^	0.05	0.175	0.500	0.01	0.300	0.353	0.15	**0.004**	0.133	0.13	0.137	0.068
${\mathrm{NO}}_{3}^{-}$	mg kg^−1^	0.05	**0.012**	0.091	0.04	**0.002**	0.096	0.37	**0.001**	**0.005**	0.11	**0.024**	**0.005**
C:N ratio	unitless	0.00	0.656	0.614	0.00	0.270	0.972	0.73	**0.001**	0.53	0.04	0.130	0.624
BD	g cm^−3^	0.00	0.835	0.685	0.00	0.292	0.416	0.80	**0.001**	0.195	0.01	0.894	0.328
TOC	%	0.01	**0.003**	0.274	0.00	0.756	0.439	0.93	**0.001**	**0.001**	0.01	**0.044**	**0.041**
TN	%	0.02	**< 0.001**	0.223	0.00	0.824	0.559	0.90	**0.001**	**0.001**	0.02	**0.004**	0.162
pH	H_2_O	0.39	**< 0.001**	0.322	0.14	0.001	0.468	0.27	**0.001**	0.052	0.07	**0.002**	0.699
*16S*	Gene copies per g soil	0.09	**0.001**	0.656	0.00	0.332	0.649	0.52	**0.001**	0.108	0.11	**0.003**	0.136
*amoA* (AOA)	Gene copies per g soil	0.08	**0.003**	0.570	0.04	**0.009**	0.097	0.48	**0.001**	**0.009**	0.08	**0.030**	0.197
*amoA* (AOB)	Gene copies per g soil	0.14	**0.001**	0.601	0.00	0.472	**0.038**	0.37	**0.001**	**0.006**	0.10	**0.026**	0.280
*nifH*	Gene copies per g soil	0.02	0.127	0.584	0.02	**0.042**	0.659	0.55	**0.001**	0.107	0.08	**0.028**	0.690
*nirK*	Gene copies per g soil	0.05	**0.005**	0.369	0.01	0.222	0.885	0.62	**0.001**	0.090	0.07	**0.043**	0.244
*nirS*	Gene copies per g soil	0.09	**0.002**	0.308	0.00	0.413	0.735	0.36	**0.001**	0.606	0.08	0.159	0.330
*nosZI*	Gene copies per g soil	0.11	**0.001**	0.405	0.01	0.251	0.767	0.47	**0.001**	**0.032**	0.12	**0.001**	0.430

*Note:* Results from full factorial analysis showing the most relevant factors based on variance explained (*R* ^2^). Each variable was tested separately using PERMANOVA to test for differences between groups and PERMDISP to test for homogeneity of multivariate dispersion. *p*‐values < 0.05 (shown in bold) indicate significant effects. Variables include soil chemical properties (${\mathrm{NH}}_{4}^{+}$, ${\mathrm{NO}}_{3}^{-}$, C:N ratio, bulk density [BD], total organic carbon [TOC], total nitrogen [TN], pH) and functional gene abundances (*16S, amoA* [AOA], *amoA* [AOB], *nifH, nirK, nirS, nosZI*) measured as gene copies per gram soil. *p*‐values for PERMANOVA are shown as P, while *p*‐values for PERMDISP are shown as D. T = organic resource treatment, N = Mineral N application, S = site.

Abbreviations: 16S, 16S rRNA gene (total bacteria); *amoA* (AOA), ammonia‐oxidizing archaea; *amoA* (AOB), ammonia‐oxidizing bacteria; BD, bulk density; C:N ratio, carbon to nitrogen ratio; NH_4_, ammonium; nifH, nitrogen‐fixing bacteria; nirK, nitrite‐reducing bacteria; nirS, nitrite‐reducing bacteria; NO_3_, nitrate; nosZ1, nitrous oxide‐reducing bacteria; TN, total nitrogen; TOC, total organic carbon.

**FIGURE 2:**
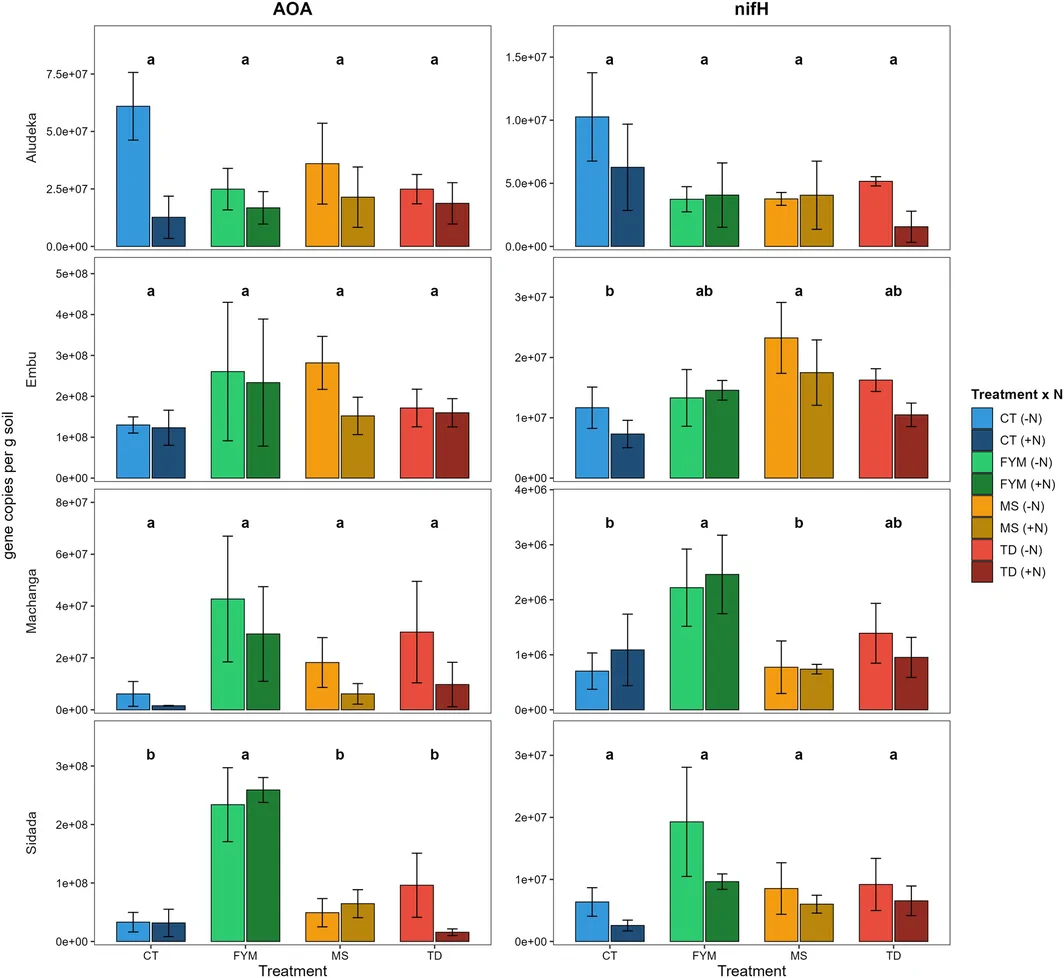
N‐cycling functional gene abundance across organic resource treatments (treatment) and sites with and without mineral N addition (N). Barplots show gene copies per gram soil for ammonia‐oxidizing archaea (AOA) and nitrogen‐fixing bacteria (nifH) across four sites (Aludeka, Embu, Machanga, Sidada) and four organic resource treatments (CT = control, without organic inputs, FYM = farmyard manure, MS = maize ( *Zea mays* ) stover, TD = *Tithonia diversifolia* ). Dark colors represent nitrogen addition (+N) and light colors represent no nitrogen addition (−N). Different letters indicate significant differences between treatments (pairwise PERMANOVA, *p* < 0.05).

Organic amendments produced contrasting site‐specific responses for both genes (Figure 2). Ammonia‐oxidizing archaea abundances showed differentiation due to organic resource management only at Sidada, where farmyard manure resulted in substantially higher values (2.46 × 10^8^ gene copies g^−1^, 662% higher than control at 3.23 × 10^7^ gene copies g^−1^) compared to other treatments (3.23–5.69 × 10^7^ gene copies g^−1^), while Aludeka, Embu, and Machanga exhibited no clear effects of organic resource management (Figure S3b). Nitrogen‐fixing bacteria exhibited strong organic resource management effects at Embu and Machanga but not at Aludeka or Sidada (Figure S3d). At Embu, maize stover produced the highest *nifH* abundances (2.04 × 10^7^ gene copies g^−1^, 115% higher than control at 9.49 × 10^6^ gene copies g^−1^), while at Machanga, farmyard manure significantly elevated *nifH* abundance (2.34 × 10^6^ gene copies g^−1^, 161% higher than control at 8.96 × 10^5^ gene copies g^−1^).

Ammonia‐oxidizing bacteria (bacterial *amoA*) showed the strongest organic resource response among all functional genes (Table 2), although this response was highly site‐dependent. Overall, farmyard manure produced the highest bacterial *amoA* abundance (1.0 × 10^6^ gene copies g^−1^, 621% higher than the control at 1.4 × 10^5^ gene copies g^−1^), whereas Tithonia and maize stover showed smaller increases (229% and 125%, respectively) (Figure S3c). This response was driven almost entirely by Machanga, where farmyard manure significantly increased bacterial *amoA* abundance (2.6 × 10^5^ gene copies g^−1^, 2920% higher than the control at 8.8 × 10^3^ gene copies g^−1^; Table S4), while maize stover and Tithonia remained low and did not differ significantly from the control (2.2 × 10^4^ and 5.1 × 10^4^ gene copies g^−1^). No significant treatment differentiation was observed at Aludeka, Embu, or Sidada; although Sidada showed high overall bacterial *amoA* abundances, treatment differences there were not significant owing to high variability among replicates (Figure S3c).

Other N‐cycling genes showed similar patterns, with farmyard manure consistently producing the highest abundances. Among denitrification genes, *nosZI* and *nirS* responded strongly to farmyard manure, with 196% and 326% increases over control, respectively. Organic resource treatment differentiation occurred exclusively at Machanga, where farmyard manure produced substantially higher abundances for *nosZI* (1.34 × 10^7^ gene copies g^−1^, 1193% above control) and *nirS* (3.07 × 10^6^ gene copies g^−1^, 997% above control) compared to other treatments (Figure S3f,g). The *nirK* gene followed the same pattern, increasing by 436% with farmyard manure compared to the control at Machanga (3.34 × 10^8^ vs. 6.23 × 10^7^ gene copies g^−1^) but showing no treatment differentiation at other sites (Figure S3e).

Total prokaryotes (16S rRNA) exhibited organic resource treatment effects that varied by site. At Embu, maize stover produced the highest abundances (3.54 × 10^9^ gene copies g^−1^, 128% above control), while at Machanga and Sidada, farmyard manure treatment showed the highest abundances (6.23 × 10^8^ and 2.98 × 10^9^ gene copies g^−1^, representing 535% and 310% increases, respectively) (Figure S3a).

Mineral N fertilization had a narrow, functionally specific effect. Among all measured genes it significantly reduced only ammonia‐oxidizing archaea (archaeal *amoA*), by 23% (+N: 7.23 × 10^7^ vs. −N: 9.37 × 10^7^ gene copies g^−1^), and nitrogen‐fixing bacteria (nifH), by 29% (+N: 5.99 × 10^6^ vs. −N: 8.49 × 10^6^ gene copies g^−1^), each explaining 2%–4% of variance (Table 2). It did not significantly alter overall prokaryotic or fungal α‐ and β‐diversity, indicating a targeted suppression of N‐cycling functional groups rather than a community‐wide shift.

### 3.2 Microbial α‐Diversity

**FIGURE 3:**
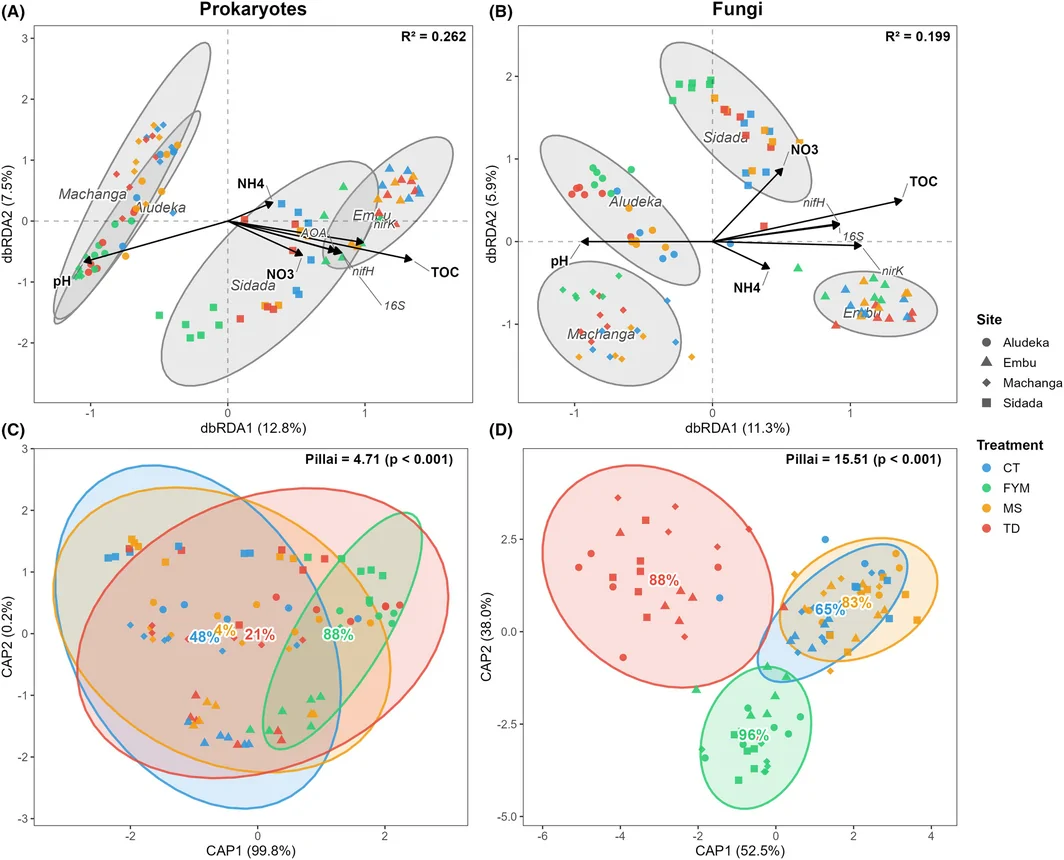
Prokaryotic and fungal community beta diversity patterns across organic resource treatments and sites revealed through distance‐based redundancy analysis (dbRDA) constrained by environmental variables (A, B) and through canonical analysis of principal coordinates (CAP) constrained by organic resource treatment (C, D) for prokaryotic (A, C) and fungal (B, D) communities. Points represent individual samples color‐coded by organic resource treatment (CT = control without organic inputs, FYM = farmyard manure, MS = maize stover, TD = *Tithonia diversifolia* ) and symbol‐coded by site (circles = Aludeka, triangles = Embu, diamonds = Machanga, squares = Sidada). (A, B) Grey ellipses represent site groupings with 95% confidence interval. Black arrows indicate environmental variables showing significant (*p* < 0.05) associations with community structure. *R* ^2^ values show the proportion of variance explained by selected environmental variables. (C, D) Colored ellipses represent treatment groupings with 95% confidence interval. CAP reclassification success rates (%) within each treatment ellipse provide quantitative estimation of discrimination between treatments. Pillai's trace test statistics with *p*‐values indicate overall effect size.

For prokaryotes, site (16%–19%), organic amendment treatments (15%–22%), and their interactions (11%–19% for evenness and Shannon diversity) collectively explained variance in α‐diversity, while for fungi, the dominant driver varied by metric: site controlled richness (26%), organic amendments dominated evenness and Shannon diversity (18%–19%), and Treatment × Site interactions were consistently important across all metrics (11%–14%) (Table 3). Mineral N fertilization had minimal effects on both prokaryotes (3%–7% of variance) and fungi (0%–5% of variance), with heterogeneous dispersion for prokaryotic evenness and Shannon diversity warranting cautious interpretation. Full factorial analysis revealed that *Treatment* by *Site* interactions explained more variance (8%–19% of variance) compared to other possible two‐way or three‐way interactions. Given the minimal effects of mineral N fertilization and dispersion heterogeneity, mineral N input was excluded from β‐diversity visualizations (Figure 3).

**TABLE 3:** Effects of organic resource treatment, mineral N application, site, and their interactions on prokaryotic and fungal α‐diversity and β‐diversity assessed by permutational analysis of variance (PERMANOVA) and permutational analysis of multivariate dispersion (PERMDISP).

	α‐Diversity									β‐Diversity		
	Observed richness (S)			Pielou's evenness (J)			Shannon diversity (H)			Bray–Curtis dissimilarity		
	*R* ^2^	FM (*p*)	FD (*p*)	*R* ^2^	FM (*p*)	FD (*p*)	*R* ^2^	FM (*p*)	FD (*p*)	*R* ^2^	FM (*p*)	FD (*p*)
Prokaryotes												
OR Treatment (T)	0.20	13.32	0.74	0.15	9.26	0.24	0.22	15.20	0.83	0.08	4.54	1.12
		**(0.001)**	(0.542)		**(0.001)**	(0.884)		**(0.001)**	(0.499)		**(0.001)**	(0.349)
Mineral N application (N)	0.07	14.63	0.8	0.03	5.02	4.65	0.06	12.25	9.33	0.03	4.75	3.87
		**(0.001)**	(0.388)		**(0.030)**	**(0.032)**		**(0.001)**	**(0.003)**		**(0.001)**	(0.060)
Site (S)	0.19	12.75	0.57	0.16	9.84	1.52	0.18	12.19	2.70	0.30	18.03	12.57
		**(0.001)**	(0.652)		**(0.001)**	(0.219)		**(0.001)**	(0.053)		**(0.001)**	**(0.001)**
T × N	0.09	5.65	0.32	0.01	0.80	1.09	0.04	3.12	0.99	0.03	1.62	0.62
		**(0.002)**	(0.938)		(0.491)	(0.362)		**(0.025)**	(0.419)		**(0.004)**	(0.727)
S × T	0.08	1.67	0.81	0.19	3.99	1.30	0.11	2.47	1.32	0.12	2.34	1.7
		(0.105)	(0.683)		**(0.002)**	(0.230)		**(0.017)**	(0.186)		**(0.001)**	(0.070)
S × N	0.03	1.67	0.34	0.08	4.72	1.22	0.06	4.48	1.76	0.03	1.96	4.92
		(0.173)	(0.935)		**(0.008)**	(0.290)		**(0.005)**	(0.111)		**(0.001)**	**(0.001)**
T × N × S	0.02	0.34	0.54	0.04	0.84	0.86	0.03	0.58	0.75	0.06	1.20	0.91
		(0.966)	(0.965)		(0.564)	(0.669)		(0.788)	(0.811)		(0.061)	(0.600)
Fungi												
OR Treatment (T)	0.04	1.96	0.37	0.19	8.86	0.65	0.18	8.45	0.97	0.09	5.31	0.49
		(0.084)	(0.768)		**(0.001)**	(0.582)		**(0.001)**	(0.451)		**(0.001)**	(0.686)
Mineral N application (N)	0.00	0.67	0.04	0.05	7.14	0.00	0.05	6.87	0.04	0.01	2.21	0.33
		(0.400)	(0.834)		**(0.005)**	(0.963)		**(0.006)**	(0.873)		**(0.005)**	(0.577)
Site (S)	0.26	12.17	0.17	0.05	2.10	2.03	0.10	4.81	1.65	0.28	15.94	3.00
		**(0.001)**	(0.924)		(0.105)	(0.098)		**(0.004)**	(0.183)		**(0.001)**	**(0.038)**
T × N	0.06	2.72	0.55	0.02	0.76	1.06	0.01	0.33	1.03	0.02	1.25	0.16
		**(0.027)**	(0.773)		(0.530)	(0.396)		(0.800)	(0.404)		(0.094)	(0.994)
S × T	0.11	1.80	0.83	0.13	1.94	0.68	0.14	2.25	1.14	0.13	2.47	1.67
		**(0.034)**	(0.649)		**(0.047)**	(0.818)		**(0.032)**	(0.339)		**(0.001)**	(0.079)
S × N	0.03	1.54	0.40	0.01	0.29	1.07	0.01	0.57	0.76	0.02	1.21	1.88
		(0.166)	(0.907)		(0.840)	(0.337)		(0.618)	(0.626)		(0.116)	(0.092)
T × N × S	0.04	0.67	0.44	0.09	1.32	0.54	0.07	1.15	0.60	0.05	0.98	0.71
		(0.595)	(0.994)		(0.235)	(0.982)		(0.328)	(0.951)		(0.599)	(0.873)

*Note:* α‐diversity was measured using observed richness (S), Pielou's evenness (J), and Shannon diversity (H). Beta diversity was measured using Bray‐Curtis dissimilarity. Values indicate the explained variance (*R* ^2^), *F*‐ratio (*F*), and significance level (*p*) for PERMANOVA [FM (*p*)] and PERMDISP [FD (*p*)]. Significant results (*p* < 0.05) are indicated with bold numbers. Organic resource treatments: CT = control without organic inputs, FYM = farmyard manure, MS = maize (*Zea mays*) stover, TD = *Tithonia diversifolia*. Mineral N application: with (+N) and without (−N) nitrogen addition. Sites: Aludeka, Embu, Machanga, Sidada. T = organic resource treatment, *N* = Mineral N application, S = site.

Overall, farmyard manure resulted in the highest prokaryotic richness (S = 11,500, 53% above control), while maize stover was associated with lower values (S = 10,000, 33% above control), and the control had the lowest richness (S = 7500). prokaryotic richness under Tithonia did not differ from farmyard manure or maize stover amendments (Figure S4a). At Embu, both farmyard manure and maize stover were associated with increased richness, while at Machanga, all three organic amendments (farmyard manure, maize stover, and Tithonia) resulted in higher richness than control. At Aludeka and Sidada, only farmyard manure had higher richness than the control. In contrast, fungal richness was not different between treatments (S = 800–950) and remained statistically similar across treatments at most sites (Figure S5a). Treatment effects were absent at Embu and Sidada, while at Machanga all three amendments were elevated above control, and at Aludeka farmyard manure differed from Tithonia but not from control (Figure S5a).

Overall, farmyard manure and Tithonia resulted in the highest prokaryotic evenness (J = 0.85 and 0.83, representing 8% and 5% above control at J = 0.79, respectively) (Figure S4b). Treatment differentiation varied by site. Aludeka had the same trend as observed overall, while at Machanga and Sidada, farmyard manure was associated with the highest values, with control, maize stover, and Tithonia lower, whereas at Embu, no treatment differentiation was observed. In contrast, fungal evenness had a distinct pattern, with Tithonia resulting in the lowest overall evenness (J = 0.61), approximately 10% lower than the other treatments (J = 0.67–0.68) (Figure S5b). Tithonia was associated with site‐specific responses, with values lower than farmyard manure and maize stover and similar to the control at Aludeka, lower than both control and maize stover at Embu, consistent with overall trends at Sidada, and resulted no differences among treatments at Machanga.

Overall, farmyard manure resulted in the highest prokaryotic Shannon diversity (H = 7.9, 10% above control at H = 7.2). Tithonia also had increased diversity (H = 7.7, 7% above control) (Figure S4c), and maize stover had intermediate values (H = 7.4, 3% above control) (Figure S4c). Treatment differentiation varied by site. At Aludeka, Tithonia and farmyard manure resulted in the highest Shannon diversity compared to control. Likewise, at Machanga, farmyard manure, Tithonia, and maize stover all resulted in higher diversity than control. At Sidada, only farmyard manure was elevated above all other treatments, whereas Embu resulted in no treatment differentiation. In contrast, an overall pattern was observed for fungal Shannon diversity, with Tithonia lowest (H = 4.1, approximately 9%–11% lower than other treatments at H = 4.5–4.6) (Figure S5c). This overall trend was most evident at Embu and Sidada, where Tithonia had the lowest diversity and differed significantly from other treatments. At Aludeka, Tithonia was lower than farmyard manure and maize stover, while control had no clear differentiation. However, at Machanga, the pattern reversed, with control having the lowest diversity compared to Tithonia and farmyard manure.

### 3.3 Microbial β‐Diversity

Site, Org Res *Treatment* × *Site* interactions, and organic amendment management collectively explained the majority of variance in both prokaryotic (50% total: 30%, 12%, and 8%, respectively) and fungal (50% total: 28%, 13%, and 9%, respectively) β‐diversity, while mineral N fertilization resulted in minimal effects (3% and 1%) (Table 3). Heterogeneous dispersion was observed for site effects in both prokaryotes and fungi, and for Site by mineral N input interactions in prokaryotes, warranting cautious interpretation. Full factorial analysis revealed that *Treatment* by *Site* interactions explained more variance compared to other possible two‐way or three‐way interactions. These Treatment × Site interactions were consistent across response types, being concentrated at Machanga for the functional genes, while prokaryotic diversity responded to farmyard manure at all four sites and fungal diversity responded mainly to *T. diversifolia* at the clay‐rich sites (Table S5). Given the limited variance explained by mineral N fertilization and dispersion heterogeneity in nitrogen‐related interactions, this factor was excluded from the visualization exploring drivers of community structure.

Environmental variables explained substantial variation in both prokaryotic and fungal community composition, accounting for the strong site effects observed in β‐diversity patterns (Figure 3A,B). For prokaryotes, distance‐based redundancy analysis revealed that TOC and pH were the primary drivers of community structure, with soil properties and functional gene abundances included as constraining variables, collectively explaining 26% of variance (Figure 3A). Similarly, fungal communities were shaped predominantly by TOC, pH, and nitrate (${\mathrm{NO}}_{3}^{-}$), with these factors together explaining 20% of the variance (Figure 3B). Functional gene abundances co‐varied with TOC in both prokaryotic and fungal ordinations, reflecting their shared association with the site‐level carbon gradient rather than a causal influence on community composition, while pH showed the inverse pattern. Overall, these environmental gradients corresponded strongly with site‐level differences, with samples clustering distinctly by site along the environmental vectors. TOC and pH co‐varied with soil texture, with clayey sites (Embu and Sidada) characterized by relatively higher TOC and lower pH values, as compared to sandy sites (Machanga and Aludeka). Site‐specific db‐RDA analyses confirmed varying environmental drivers across sites (Figures S4e and S5e).

Despite strong, site‐dependent environmental control over community composition, organic amendment treatments resulted in distinguishable community groupings when constrained by treatment (Figure 3C,D). Canonical analysis of principal coordinates discriminated prokaryotic communities by treatment (Pillai's trace = 4.71), indicating that Org Res treatments generated distinct microbial assemblages beyond site‐driven environmental variation (Figure 3C). Particularly, farmyard manure separated in community structure from control assemblages, achieving reclassification success rates of 88%. Fungal communities showed stronger treatment discrimination (Pillai's trace = 15.51), with farmyard manure and Tithonia primarily differentiating from control assemblages, achieving reclassification success rates of 96% and 88%, respectively (Figure 3D). Treatment discrimination was significant at individual sites for both prokaryotic and fungal communities, with even clearer separation patterns observed in site‐specific analyses (Figures S4d and S5d).

### 3.4 Microbial Taxonomic Composition

**FIGURE 4:**
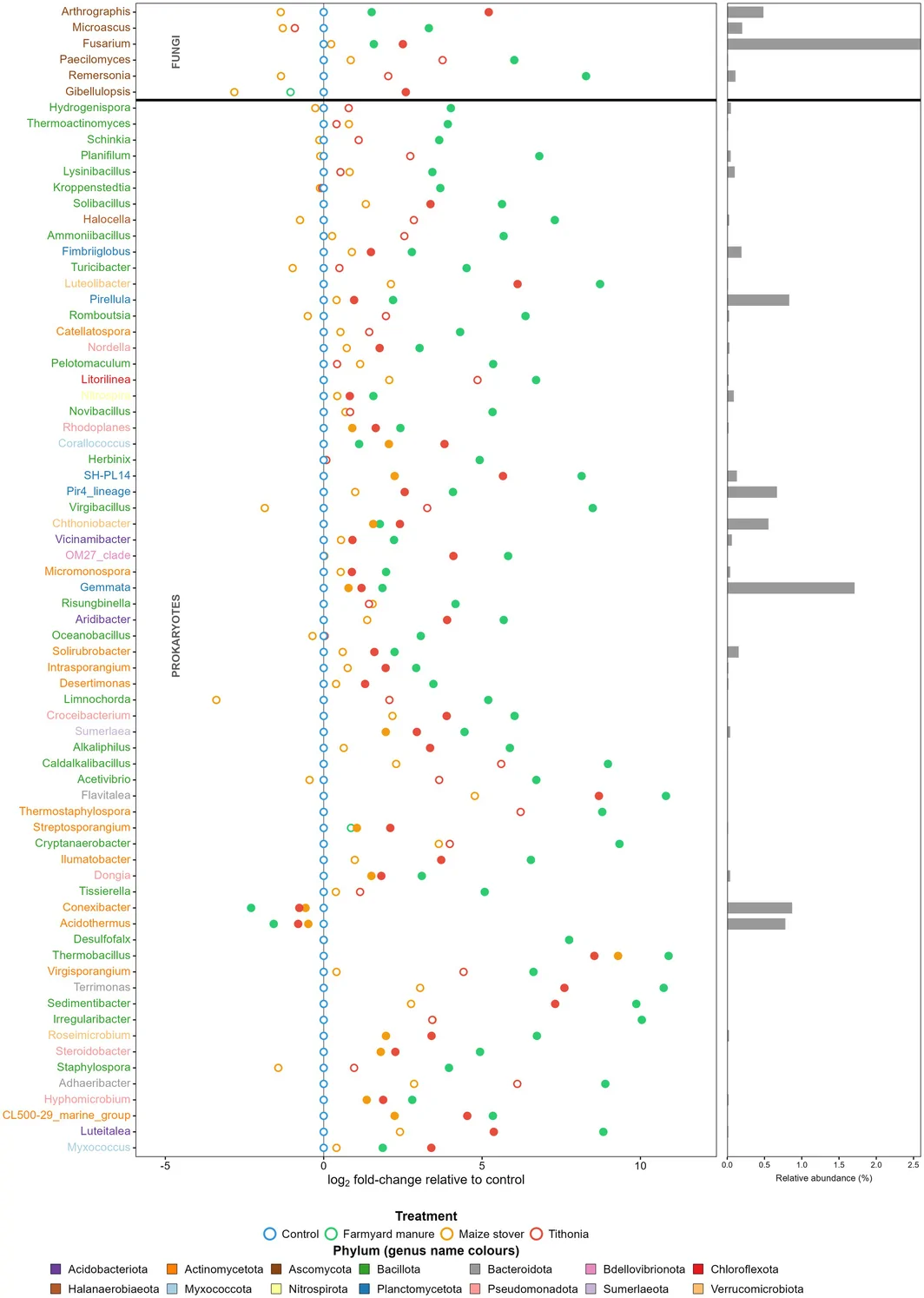
Log2 fold‐change relative to the control for prokaryotic genera (*n* = 66, bottom section) and fungal genera (*n* = 6, top section) selected based on strong statistical effects (*F* ≥ 10, FDR‐adjusted *p* < 0.05) following organic amendment treatments compared to control (without organic inputs). Genera are ordered by *F* effect size within each domain (most responsive at top of each section) and colored by phylum. Black horizontal line separates prokaryotic and fungal communities. Treatments indicated by circles: Control (blue), farmyard manure (green), maize stover (orange), *Tithonia diversifolia* (red). Filled circles represent genera significantly different from control (pairwise PERMANOVA, *p* < 0.05); hollow circles represent non‐significant differences. Control is always hollow as the reference baseline. Right panel shows mean relative abundance percentages. Statistical analysis based on PERMANOVA (adonis2, 999 permutations) with Benjamini‐Hochberg procedure for false discovery rate control (*p* < 0.05).

After correction for multiple testing (Benjamini‐Hochberg), 66 prokaryotic genera across 13 phyla were observed to be strongly (*F* ≥ 10) and significantly (PERMANOVA; adjusted *p* < 0.05) influenced by organic amendments. These genera were visualized based on descending F‐ratios (Figure 4). Pairwise PERMANOVA comparisons (each treatment vs. control) revealed that farmyard manure significantly altered 65 of the 66 genera (98.5%), while *Tithonia* and maize stover affected 37 (56.1%) and 15 (22.7%) genera, respectively. After correction for multiple testing (Benjamini‐Hochberg), only 6 fungal genera from the phylum Ascomycota exhibited strong (*F* ≥ 10) and significant (adjusted *p* < 0.05) responses to organic amendments (Figure 4, top section). Pairwise PERMANOVA revealed farmyard manure significantly affected 5 of 6 genera (83.3%), Tithonia affected 3 genera (50%), while maize stover showed no significant effects on any fungal genus.

Prokaryotic genera under farmyard manure showed the strongest enrichment relative to control. *Bacillota* dominated the most responsive genera, with *Hydrogenispora, Thermoactinomyces*, *Schinkia*, *Planifilum*, *Lysinibacillus*, *Kroppenstedtia*, *Solibacillus* and *Ammoniibacillus* all having increased relative abundance under farmyard manure compared to control (Figure 4). Among the top‐ranked responsive genera, additional phyla included Halanaerobiaeota (*Halocella*) and Planctomycetota (*Fimbriiglobus*), which similarly increased in relative abundance under farmyard manure. While these top responders had consistent enrichment under farmyard manure, *Tithonia* and maize stover also increased most of these genera, though less strongly and less frequently than farmyard manure. Only two genera, *Conexibacter* and *Acidothermus*, decreased significantly, and did so under all three amendments. Lower‐ranking genera across additional phyla exhibited comparable treatment responses (Figure 4).

Fungal genera revealed limited taxonomic breadth compared to prokaryotic communities (Figure 4). The six responsive genera, in order of decreasing F‐ratio, included *Arthrographis*, *Microascus*, *Fusarium*, *Paecilomyces*, *Remersonia*, and *Gibellulopsis*. Farmyard manure increased the relative abundance of *Arthrographis, Microascus, Fusarium, Paecilomyces* and *Remersonia* compared to the control, whereas *Gibellulopsis* was not significantly affected. *Tithonia* increased *Arthrographis*, *Fusarium* and *Gibellulopsis*. Maize stover did not significantly differ from the control.

## 4 Discussion

### 4.1 Long‐Term Agricultural Management Drives Soil Microbial Community Structure and Functionality Within Sites

Site‐dominated variations in microbial communities are consistent with continental‐ and global‐ scale patterns, where edaphic properties, particularly pH, act as primary environmental filters controlling microbial biogeography (Cowan et al. 2022; Fierer and Jackson 2006). Site effects overshadowed management across the four long‐term trials, explaining substantial portions of the variance in soil properties, functional genes, and community composition. Despite this strong environmental filtering, sustained organic amendments created distinguishable effects on microbial community composition across all sites, with farmyard manure achieving particularly strong discrimination for both prokaryotes and fungi. This capacity for long‐term management to shape communities within the constraints of site‐specific pedoclimatic conditions represents an advancement in knowledge over shorter‐term East African studies (duration: 2 years), where weak treatment effects manifested only at the genus level without restructuring overall community composition (Köberl et al. 2022), and over Zambian trials (duration: 126 days), where inherent soil properties constrained organic amendment responses to only one of two contrasting sites (Hamamoto et al. 2022). The results from our two‐decade old experiments demonstrate that sustained organic resource inputs generate predictable selection pressures that are strong enough to produce a visible effect despite environmental filtering (Zhou and Ning 2017; Stegen et al. 2012). This provides evidence that management can determine tropical soil microbiomes when long‐term maintenance is performed across climatically and edaphically heterogeneous landscapes.

Although long‐term mineral N application explained limited variation in our data, the consistent capacity of farmyard manure to simultaneously alter soil properties, functional gene abundances, and community composition across all four heterogeneous agroecological sites in Kenya may reflect its multifunctional properties. Farmyard manure provides a balanced mix of labile and recalcitrant carbon substrates and nitrogen, including readily decomposable compounds and lignified materials, which stimulate copiotrophic taxa and nitrogen‐cycling guilds (Ding et al. 2016; Francioli et al. 2016; Levy‐Booth et al. 2014). Additionally, farmyard manure progressively modifies soil physical structure through aggregate formation (Bulluck et al. 2002; Six et al. 2000) and organic matter accumulation (Liang et al. 2017), which in turn alters pH buffering capacity (Bulluck et al. 2002), cation exchange (Hati et al. 2007), and nutrient stoichiometry (Fanin et al. 2013). Unlike plant‐based amendments, such as *T. diversifolia* , which is typically sourced locally and contributes only aboveground material, farmyard manure introduces substantial and diverse microbial inocula from animal digestive systems that directly contribute to soil microbial diversity (Liu et al. 2020; Hartmann et al. 2015; Sun et al. 2015). Soil conditions appear to play a role, as TOC and pH were the primary constraints on community structure, but several of the genera enriched by farmyard manure are gut‐associated (*Romboutsia, Turicibacter*), thermophilic (*Thermoactinomyces, Planifilum*) or obligately anaerobic taxa (*Hydrogenispora, Halocella*), which would not be expected to establish in aerated topsoil from the resident community, suggesting some contribution of immigration with the amendment. These interacting mechanisms seem to create cascading effects that, over time, generate more diverse ecological niches within the soil matrix, thereby buffering communities against site‐specific environmental stresses compared to the other organic amendments tested in this study. The temporal requirement for these cascading effects may explain why short‐term Zambian trials showed amendment responses constrained by inherent soil texture and water‐holding capacity, affecting only microbial abundance without restructuring communities (Hamamoto et al. 2022). In contrast, our decadal application enabled cumulative improvements in soil properties, effectively building different habitats over time through changes in TOC, pH, and soil structure. These progressive modifications drove shifts in both functional potential and taxonomic composition. These patterns suggest that temporal duration and the progressive modification of soil properties, rather than just immediate organic resource quality, determine whether organic amendments can generate predictable changes across the soil property‐gene‐community continuum in environmentally variable tropical (smallholder) agroecosystems. These findings support our first hypothesis that farmyard manure distinctly shapes communities across sites.

### 4.2 Site‐Specific Functional Responses Highlight Where Soil Health Management Matters Most

While many studies demonstrate the importance of mineral N application for crop productivity in sub‐Saharan Africa (Tully et al. 2015; Bekunda et al. 2010), our results show it had minimal impact on soil microbial communities compared to organic amendments. However, this contrast must be interpreted considering that organic inputs persist in soil as carbon substrates, while mineral nitrogen is rapidly consumed or lost, fundamentally differing in their capacity to shape microbial communities. Nevertheless, because mineral N increased crop biomass and residue return in these trials (Laub, Corbeels, Ndungu, et al. 2023), some indirect influence on microbial communities might be expected, which makes its limited direct effect notable. Our findings nonetheless expose a trade‐off central to sustainable intensification: mineral nitrogen supports crop productivity but contributes little to soil biological health, whereas organic amendments build microbial function and multifunctionality yet, as shown below, do not always raise yield (Creamer et al. 2022). Although farmyard manure consistently altered microbial communities across all sites, the magnitude of functional responses varied substantially with the inherent soil properties of different agroecological sites. Most notably, at Machanga, farmyard manure markedly increased functional gene abundances (Figure S3a–g), with proportional increases far exceeding those at the other sites. This appears to reflect more than textural differences, as Aludeka has a comparable texture and bulk density but showed no significant gene response. Machanga combines coarse texture with the lowest organic carbon and nitrogen (3.52 and 0.522) and by far the lowest rainfall (795 mm), so that the same absolute increment acts on a much lower microbial baseline. Nonetheless, these enhanced microbial functional potentials did not translate to yield increases, as rainfall limitation constrained crop productivity despite elevated soil fertility and microbial activity (Laub et al. 2025). This disconnect demonstrates that under severe water stress, microbial functional capacity responds to substrate addition independently of agronomic outcomes, with nutrient availability exceeding what moisture‐limited crops can utilize. In contrast, Aludeka showed comparably strong diversity responses to farmyard manure, is not moisture‐limited, suggesting that the enhanced microbial functional potential can translate to agronomic benefits where water availability does not constrain crop growth. Furthermore, the differential responsiveness of coarse‐textured soils to organic amendments is consistent with the conceptual framework of Hartmann and Six (2023), proposing that sandy soils with lower “baseline” organic carbon and reduced nutrient buffering capacity exhibit stronger microbial responses to substrate additions through enhanced aggregate formation and increased availability of labile carbon sources that favor copiotrophic microbial growth (Francioli et al. 2016; Six et al. 2006). Similar patterns of stronger microbial responses to organic amendments in coarse‐textured, low organic carbon soils have been documented in Zambia (Hamamoto et al. 2022), Burkina Faso (Diallo‐Diagne et al. 2016), and India (Aparna et al. 2014).

In our study, the clustering of functional gene abundances with total organic carbon along the clayey–sandy soil gradient likely reflects the combined effects of texture and climate that define site‐level differences, rather than organic matter effects per se, which are better demonstrated through within‐site treatment responses (Figures S4e and S5e). Organic C serves as both an energy source and a structural component that modulates microbial habitat availability, with higher total organic C soils providing more diverse microhabitats, greater moisture retention, and more stable carbon substrates that support the maintenance of larger functional gene pools (Trivedi et al. 2016; Fierer et al. 2012). The positive association between functional gene abundances and total organic C across sites reflects the confounding effects of texture, climate, and organic matter that cannot be separated from the four sites. This pattern is consistent with the principle that microbial functional capacity, encoded in genes for N cycling, C decomposition, and nutrient acquisition, requires sustained energy investment that is only feasible when C availability exceeds a threshold necessary to support gene expression and enzyme production (Clark et al. 2021; Schimel and Schaeffer 2012). More conclusively, within‐site comparisons showed that in sandy soil, with low total organic C treatments, limited C availability constrains microbial biomass and the energetically costly processes of maintaining and expressing heterotrophic functional genes (Figures S4e and S5e). However, autotrophic processes such as ammonia oxidation are not directly limited by C availability but may still respond indirectly to organic amendments through improved soil structure, moisture retention, and nutrient availability. Together, these conditions create an environment where functional potential remains latent until substrate additions trigger both population growth and metabolic activation (Hartmann and Six 2023; Blagodatskaya and Kuzyakov 2013).

Mineral N fertilization showed contrasting effects on functional gene abundances, with reduced abundances of ammonia‐oxidizing archaea and nitrogen‐fixing bacteria under +N conditions, likely reflecting reduced selective pressure for these energetically costly processes when mineral N is readily available (Geisseler and Scow 2014; Verhamme et al. 2011; Reed et al. 2011). However, mineral N had substantially less influence on microbial communities than organic resources, except for N‐cycling microbes, which is consistent with its limited effect on soil organic C in these trials (Laub, Corbeels, Couëdel, et al. 2023). The impact of fertilizer practices on (biological) soil health via biomass productivitydepends on multiple interacting factors such as soil texture, nutrient imbalances, and environmental conditions including rainfall (Roobroeck et al. 2021; Kibunja et al. 2012). This differential response likely reflects that mineral fertilizers provide substrates without the complementary C sources and physical habitat modifications, such as improved soil aggregation and pore network connectivity, that simultaneously stimulate diverse functional guilds under organic amendment (Francioli et al. 2016; Hartmann et al. 2015). Hence, sole reliance on mineral N fertilizers without integrating organic amendments or other soil health practices can therefore lead to a lower microbial biomass and overall soil fertility (Adams et al. 2020; Kibunja et al. 2012). Future research should prioritize understanding the interactive effects of fertilizer management with other agronomic practices, particularly crop diversification strategies such as intercropping and rotation systems, to prevent long‐term C decline, promote soil health and in particular functional redundancy in tropical agricultural systems (Laub et al. 2024). The differential functional responses refine our first hypothesis: while farmyard manure altered community composition at all sites, functional gene responses were strongly site‐dependent and concentrated at the driest, most nutrient‐poor site.

Our results indicate that organic inputs yield the largest biological gains on coarse, carbon‐poor soils, but that this benefit translates into agronomic returns only where rainfall is adequate. Under moisture limitation, as at Machanga, organic amendments are therefore better regarded as long‐term investments in soil health and carbon than for immediate yield responses, and are most effective when combined with water‐conservation measures. Furthermore, mineral nitrogen alone is insufficient to support soil biological function anywhere (Vanlauwe et al. 2026).

### 4.3 Twenty Years of Organic Resource Incorporation Shows Microbial Domain‐Specific Ecological Adaptation

Fungal and prokaryotic communities differed in response patterns to organic amendments, revealing domain‐specific ecological strategies in substrate utilization. While farmyard manure restructured both domains across all sites, the mechanisms underlying these shifts differed markedly. Fungi demonstrated dual sensitivity to both Tithonia (rapid decomposition, low C:N ratio) and farmyard manure (slower decomposition, mixed labile and recalcitrant fractions) but not to maize stover, achieving stronger treatment discrimination through concentrated shifts in a narrow guild of specialized decomposers. Conversely, prokaryotes showed manure‐driven responses with weaker treatment discrimination despite taxonomically broader shifts across 66 genera (Figure 4). This divergence potentially reflects distinct life‐history strategies where fungal communities exhibit broader substrate tolerance, responding to both readily available and moderately decomposable organic inputs (Strickland and Rousk 2010; Hanson et al. 2008). In contrast, prokaryotic communities prefer predominantly highly labile substrates (Ho et al. 2017; Fierer et al. 2007), with copiotrophic taxa rapidly exploiting readily fermentable inputs through r‐selected growth strategies (Pascault et al. 2013; Fontaine et al. 2003). These domain‐level differences in resource exploitation strategies have implications for understanding carbon and nutrient cycling in tropical agroecosystems (Pawlowska 2024; Deveau et al. 2018). The temporal dynamics of organic matter decomposition depend not only on substrate chemistry but also on the metabolic capabilities within and among microbial domains, where complementary metabolic capacities determine the stability and efficiency of nutrient cycling across different stages of residue breakdown (Louca et al. 2018; Manzoni et al. 2012). This domain‐specific divergence partially supports our second hypothesis that fungi would show clearer treatment separation than prokaryotes.

Fungi can degrade complex, high‐molecular‐weight compounds that bacteria struggle to access, with different functional guilds being recruited based on substrate quality. This was evident in how the different organic amendments stimulated distinct functional guilds despite a taxonomically narrow response (only 6 genera) (Figure 4). Farmyard manure enriched saprotrophic Ascomycota genera with complementary decomposition capabilities include: *Microascus*, a thermotolerant decomposer specialized in keratin and cellulose degradation (Lim et al. 2025); *Paecilomyces*, known for cellulolytic activity and entomopathogenic properties (Li et al. 2025; Singha and Shukla 2023; Moreno‐Gavíra et al. 2021); and *Remersonia*, a saprotroph involved in plant debris decomposition and biofilm formation (Feng et al. 2023). *Tithonia* amendments also stimulated *Arthrographis*, involved in cellulose decomposition and bacterial‐fungal interactions in nutrient cycling (Deveau et al. 2018; Okeke and Obi 1993), and *Fusarium*, combining saprotrophic and pathogenic lifestyles for complex organic matter decomposition (Li et al. 2025; Nikitin et al. 2023), both of which were also enriched by farmyard manure, together with the Tithonia‐specific *Gibellulopsis*, a saprotrophic and pathogenic genus (Tian et al. 2021; Deveau et al. 2018; Frey‐Klett et al. 2011). As these genera include both saprotrophic and pathogenic members, this likely reflects substrate selectivity rather than a shift toward disease. This substrate guild matching demonstrates that fungal decomposer communities exhibit functional specialization along a resource quality gradient, with different taxonomic assemblages selected based on substrate‐specific decomposition capabilities (Deveau et al. 2018; Hanson et al. 2008). The taxonomically concentrated fungal response may suggest that keystone decomposer taxa drive community‐level functional shifts in these tropical soils, where a small number of functionally specialized fungi exert control over organic matter transformation rates despite the broader taxonomic shifts observed in prokaryotic communities.

Prokaryotic responses showed dominantly manure‐driven enrichment of copiotrophic taxa with limited sensitivity to substrate structural complexity, unlike fungal responses. Farmyard manure significantly affected 65 of the 66 responsive prokaryotic genera, predominantly enriching Bacillota, including mesophilic spore‐forming bacteria *Hydrogenispora* (anaerobic carbohydrate fermenters thriving in oxygen‐limited soil microsites; Liu et al. 2014). Thermophilic genera included *Thermoactinomyces* (filamentous, endospore‐forming bacteria widely distributed in compost and soil; Mokrane et al. 2015; Xian et al. 2015), Schinkia (aerobic, spore‐forming bacteria phylogenetically reclassified from the Bacillus genus; Gupta et al. 2020), and *Planifilum* (thermophilic, filamentous bacteria commonly isolated from compost; Han et al. 2013; Hatayama et al. 2005). Manure also enriched *Lysinibacillus* (versatile soil bacteria with bioremediation and plant growth‐promoting functions; Jamal and Ahmad 2022; Ahsan and Shimizu 2021; Failor et al. 2019). Such taxa characterize nutrient‐rich, disturbed environments (Francioli et al. 2016). Farmyard manure further enriched *Halocella* (phylum Halanaerobiaeota), obligately anaerobic, halophilic, cellulolytic bacteria adapted to hypersaline environments (Heng et al. 2019; Simankova et al. 1993) and *Fimbriiglobus* (phylum Planctomycetota), acidophilic, chitin‐degrading bacteria from wetland soils (Dedysh et al. 2020; Kulichevskaya et al. 2017). These trends suggest that repeated manure application modulates the soil microbiome through two complementary pathways: direct inoculation with gut‐ and composting‐associated taxa (e.g., *Thermoactinomyces, Planifilum, Hydrogenispor*a; Bagge et al. 2010), and creation of anoxic microhabitats enriched in fermentable substrates that favor both manure‐derived r‐strategists and resident soil taxa (e.g., *Fimbriiglobus, Halocella*; Ravin et al. 2018).

After almost 20 years of consistent management, *T. diversifolia* and *Z. mays* stover also enriched copiotrophic prokaryotic taxa, but less frequently and less strongly than farmyard manure, significantly affecting 56.1% and 22.7% of the responsive prokaryotic genera, respectively. This pattern contrasts with fungal responses, where *T. diversifolia* amendments in some cases led to specialized decomposer guilds. This divergence between prokaryotes and fungi indicates domain‐specific substrate‐decomposability thresholds: *Tithonia* stimulated fungal specialists and a broad but weaker prokaryotic response, whereas the more recalcitrant maize stover elicited no fungal response and affected the fewest prokaryotic genera, contrary to our hypothesis that its recalcitrance would favor fungal decomposers (Partey et al. 2013). These contrasting strategies underscore the need for diversified organic amendment portfolios in tropical agroecosystems, combining readily decomposable materials (e.g., manure) to fuel prokaryotic nutrient mobilization with complementary plant‐based amendments (e.g., *T. diversifolia* ) to sustain fungal‐mediated carbon stabilization and soil structure development.

### 4.4 Long‐Term Tropical Experiments Are Essential for Understanding Soil Biodiversity Responses to Management

Our study also points to clear priorities for future work. Sampling was restricted to the topsoil (0 to 15 cm) at a single time point, so the subsoil and the seasonal dynamics of the communities and functional genes remain open questions, and coupling gene abundances with in situ activity measurements would show how far functional potential is realized. Because the amendments were not sequenced, the contributions of microbial inoculation and habitat modification could not be separated, and the covariation of texture, climate, and organic carbon across only four sites can be disentangled only with wider site networks. These gaps are hard to close because comparable evidence is scarce. Global syntheses capture broad signals, such as the decline in microbial diversity under nitrogen fertilization (Yang et al. 2022), but are dominated by temperate systems and reveal little about tropical smallholder soils. Long‐term, sequencing‐based trials tracking soil biodiversity under sustained organic matter additions in the tropics remain few: our findings align with Asian rice systems where straw and manure inputs restructured microbial communities along soil carbon and pH gradients (Kumar et al. 2018). Equivalent organic‐amendment studies are almost absent for tropical South America, where long‐term experiments have measured microbial biomass rather than community composition (Silva et al. 2010; Hungria et al. 2009), leaving biodiversity responses to organic inputs largely unresolved.

## 5 Conclusion

After two decades of sustained organic resource inputs across four sites in contrasting Kenyan agroecological zones, we found that organic amendments reshaped soil microbial communities, despite strong environmental heterogeneity. While site‐dominated community variation was consistent with global patterns of edaphic control over microbial biogeography, farmyard manure created distinguishable prokaryotic and fungal communities across all sites. Similarly, prokaryotic and fungal taxa exhibited contrasting response patterns to organic resource inputs: farmyard manure enriched copiotrophic prokaryotes regardless of site, aligning with established life‐history strategies, while fungi had dual sensitivity to both readily available farmyard manure and easily degradable plant residues of *T. diversifolia* . Our study further showed that organic amendments stimulated the abundance of N‐cycling genes, particularly ammonia‐oxidizing bacteria, with disproportionately stronger functional responses at Machanga, the driest and most nutrient‐poor site, where baseline microbial capacity is lowest and organic inputs fill a larger ecological niche. Critically, mineral N fertilization alone did not restructure communities relative to unamended controls and modestly reduced specific N‐cycling gene abundance, demonstrating that organic resource management, not mineral inputs, drives long‐term microbial community development in these tropical agroecosystems. Combining readily decomposable materials such as farmyard manure with rapidly decomposable plant residues such as *T. diversifolia* may balance rapid nutrient cycling with sustained carbon accumulation, while more recalcitrant residues, such as *Z. mays* stover alone, offer limited microbial benefits over decadal timescales. These findings provide decadal‐scale evidence on microbial responses to ISFM in sub‐Saharan Africa, addressing knowledge gaps on long‐term microbiome dynamics in tropical agroecosystems.

## Author Contributions

**Anna M. Visscher:** conceptualization, software, methodology, data curation, investigation, formal analysis, visualization, writing – original draft, writing – review and editing, validation. **Monicah Wanjiku Mucheru‐Muna:** methodology, data curation, project administration, resources, writing – review and editing, funding acquisition, investigation, validation, conceptualization. **Samuel Mathu Ndungu:** writing – review and editing, methodology, conceptualization, investigation, formal analysis, project administration, resources, visualization, data curation, validation, funding acquisition, software. **Mirjam Pulleman:** conceptualization, supervision, methodology, validation, project administration, funding acquisition, investigation, visualization, writing – review and editing, resources. **Daniel Mugendi:** methodology, data curation, investigation, validation, funding acquisition, project administration, resources, writing – review and editing, conceptualization. **Moritz Laub:** conceptualization, validation, funding acquisition, project administration, writing – review and editing, visualization, resources. **Rafaela Feola Conz:** methodology, data curation, validation, formal analysis, resources. **Bernard Vanlauwe:** methodology, investigation, validation, project administration, resources, writing – review and editing, conceptualization, funding acquisition, supervision. **Johan Six:** conceptualization, methodology, investigation, validation, funding acquisition, visualization, writing – review and editing, project administration, resources, supervision. **Wycliffe Waswa:** investigation, methodology, data curation, validation, project administration, resources, writing – review and editing, conceptualization, funding acquisition. **Frank Rasche:** conceptualization, writing – review and editing, validation, project administration, resources, funding acquisition, investigation, methodology. **Martin Hartmann:** conceptualization, methodology, software, data curation, supervision, formal analysis, validation, investigation, project administration, resources, writing – review and editing, writing – original draft, visualization, funding acquisition. **Rebecca Yegon:** investigation, methodology, validation, data curation, funding acquisition, writing – review and editing, project administration, resources, conceptualization.

## Conflicts of Interest

The authors declare no conflicts of interest.

## Supporting information


**Table S1:** Primers used for quantitative PCR (qPCR) and amplicon sequencing of prokaryotic and fungal marker genes.
**Table S2:** Dry matter‐based measurements chemical characteristics and 95% confidence intervals of organic resources applied to all sites. Measurements were available from Embu and Machanga from 2002 to 2004, and all sites from 2005 to 2007 and in 2018 (Adapted from Laub, Corbeels, Ndungu, et al. 2023 Laub, Corbeels, Couëdel, et al. 2023 http://creativecommons.org/licenses/by/4.0/).
**Table S3:** qPCR standard curve parameters (slope, intercept, amplification efficiency in %, and R squared) for each target gene.
**Table S4:** Percentage change in microbial diversity indices and functional gene abundances under organic amendments relative to the unamended control, by site. FYM = farmyard manure, MS = maize stover, TD = *Tithonia diversifolia* . Values are % change of treatment means relative to control (pooled across mineral N levels and replicates).
**Table S5:** Summary of significant organic resource treatment effects within each site, for microbial diversity indices and N‐cycling functional gene abundances. Cells give the amendment(s) differing significantly from the unamended control at that site (↑ higher, ↓ lower; ns = no significant difference), based on the pairwise comparisons shown in Figures S1a–g, S2a–c and S3a–c. FYM = farmyard manure, MS = maize stover, TD = *Tithonia diversifolia* . Site clay content and mean annual rainfall are given for reference.
**Figure S1:** qPCR amplification curves (relative fluorescence units, RFU, versus cycle number) for the no‐template blank, the plasmid standard alone (plasmid in water), and the plasmid spiked into the DNA extract of each sample, with lines showing means and shaded bands the variation across replicates.
**Figure S2:** qPCR standard curves showing quantification cycle (*Cq*) versus log10 standard concentration for each target gene (16S, AOA, AOB, nifH, nirK, nirS, nosZI), with points showing measured standards and lines the linear regression per target.
**Figure S3a:** 16S abundances across organic resource treatments (treatment) and sites, with and without mineral nitrogen application (N). Barplots show total bacterial gene copies per gram soil across four sites (Aludeka, Embu, Machanga, Sidada) four organic resource treatments (CT = control, without organic inputs, FYM = farmyard manure, MS = maize ( *Zea mays* ) stover, TD = *Tithonia diversifolia* ). Dark colors represent nitrogen addition (+N) and light colors represent no nitrogen addition (−N). Different letters indicate significant differences between treatments (pairwise PERMANOVA, *p* < 0.05).
**Figure S3b:** Ammonia‐oxidizing archaea (AOA) gene abundances across organic resource treatments (treatment) and sites, with and without mineral nitrogen application (N). Barplots show total bacterial gene copies per gram soil across four sites (Aludeka, Embu, Machanga, Sidada) four organic resource treatments (CT = control, without organic inputs, FYM = farmyard manure, MS = maize ( *Zea mays* ) stover, TD = *Tithonia diversifolia* ). Dark colors represent nitrogen addition (+N) and light colors represent no nitrogen addition (−N). Different letters indicate significant differences between treatments (pairwise PERMANOVA, *p* < 0.05).
**Figure S3c:** Ammonia‐oxidizing bacteria (AOB) gene abundances across organic resource treatments (treatment) and sites, with and without mineral nitrogen application (N). Barplots show total bacterial gene copies per gram soil across four sites (Aludeka, Embu, Machanga, Sidada) four organic resource treatments (CT = control, without organic inputs, FYM = farmyard manure, MS = maize ( *Zea mays* ) stover, TD = *Tithonia diversifolia* ). Dark colors represent nitrogen addition (+N) and light colors represent no nitrogen addition (−N). Different letters indicate significant differences between treatments (pairwise PERMANOVA, *p* < 0.05).
**Figure S3d:** Nitrogen‐fixing bacteria (nifH) gene abundances across organic resource treatments (treatment) and sites, with and without mineral nitrogen application (N). Barplots show total bacterial gene copies per gram soil across four sites (Aludeka, Embu, Machanga, Sidada) four organic resource treatments (CT = control, without organic inputs, FYM = farmyard manure, MS = maize ( *Zea mays* ) stover, TD = *Tithonia diversifolia* ). Dark colors represent nitrogen addition (+N) and light colors represent no nitrogen addition (−N). Different letters indicate significant differences between treatments (pairwise PERMANOVA, *p* < 0.05).
**Figure S3e:** Nitrite‐reducing bacteria (nirK) gene abundances across organic resource treatments (treatment) and sites, with and without mineral nitrogen application (N). Barplots show total bacterial gene copies per gram soil across four sites (Aludeka, Embu, Machanga, Sidada) four organic resource treatments (CT = control, without organic inputs, FYM = farmyard manure, MS = maize ( *Zea mays* ) stover, TD = *Tithonia diversifolia* ). Dark colors represent nitrogen addition (+N) and light colors represent no nitrogen addition (−N). Different letters indicate significant differences between treatments (pairwise PERMANOVA, *p* < 0.05).
**Figure S3f:** Nitrite‐reducing bacteria (nirS) gene abundances across organic resource treatments (treatment) and sites, with and without mineral nitrogen application (N). Barplots show total bacterial gene copies per gram soil across four sites (Aludeka, Embu, Machanga, Sidada) four organic resource treatments (CT = control, without organic inputs, FYM = farmyard manure, MS = maize ( *Zea mays* ) stover, TD = *Tithonia diversifolia* ). Dark colors represent nitrogen addition (+N) and light colors represent no nitrogen addition (−N). Different letters indicate significant differences between treatments (pairwise PERMANOVA, *p* < 0.05).
**Figure S3g:** Nitrous oxide‐reducing bacteria (nosZI) gene abundances across organic resource treatments (treatment) and sites, with and without mineral nitrogen application (N). Barplots show total bacterial gene copies per gram soil across four sites (Aludeka, Embu, Machanga, Sidada) four organic resource treatments (CT = control, without organic inputs, FYM = farmyard manure, MS = maize ( *Zea mays* ) stover, TD = *Tithonia diversifolia* ). Dark colors represent nitrogen addition (+N) and light colors represent no nitrogen addition (−N). Different letters indicate significant differences between treatments (pairwise PERMANOVA, *p* < 0.05).
**Figure S4a:** Prokaryotic community α‐diversity (observed richness) across organic resource treatments and sites. Box plots show observed richness across four sites (Aludeka, Embu, Machanga, Sidada) and four treatments CT = control without organic inputs, FYM = farmyard manure, MS = maize ( *Zea mays* ) stover, TD = *Tithonia diversifolia* . The top panel shows combined data across all sites, while lower panels show site‐specific results. Different letters indicate significant differences between organic resource treatments (pairwise PERMANOVA, *p* < 0.05). Outliers are shown as individual points beyond the whiskers.
**Figure S4b:** Prokaryotic community α‐diversity (evenness) across organic resource treatments and sites. Box plots show observed richness across four sites (Aludeka, Embu, Machanga, Sidada) and four treatments CT = control without organic inputs, FYM = farmyard manure, MS = maize ( *Zea mays* ) stover, TD = *Tithonia diversifolia* . The top panel shows combined data across all sites, while lower panels show site‐specific results. Different letters indicate significant differences between organic resource treatments (pairwise PERMANOVA, *p* < 0.05). Outliers are shown as individual points beyond the whiskers.
**Figure S4c:** Prokaryotic community α‐diversity (Shannon diversity) across organic resource treatments and sites. Box plots show observed richness across four sites (Aludeka, Embu, Machanga, Sidada) and four treatments CT = control without organic inputs, FYM = farmyard manure, MS = maize ( *Zea mays* ) stover, TD = *Tithonia diversifolia* . The top panel shows combined data across all sites, while lower panels show site‐specific results. Different letters indicate significant differences between organic resource treatments (pairwise PERMANOVA, *p* < 0.05). Outliers are shown as individual points beyond the whiskers.
**Figure S4d:** Site‐specific prokaryotic community structure by organic resource treatment. Canonical analysis of principal coordinates (CAP) constraining differences in prokaryotic community structure by treatment at each site: (A) Sidada, (B) Aludeka, (C) Machanga, and (D) Embu. Points represent individual samples colored by organic treatment (red = CT control without organic input, blue = FYM farmyard manure, green = MS maize stove, purple = TD *Tithonia*). The CAP reclassification success rates providing a quantitative estimation of the degree of discrimination between treatments are shown within ellipses for each treatment group. The CAP equivalent to Pillai's trace test (with *p*‐value in brackets) indicating the overall effect size is provided at the top of each plot.
**Figure S4e:** Site‐specific prokaryotic community structure across organic resource treatments revealed through distance‐based redundancy analysis (db‐RDA), shown for prokaryotic communities at four sites: Aludeka, Embu, Machanga, and Sidada. Points represent individual samples colored by treatment (CT = control, FYM = farmyard manure, MS = maize stover, TD = *Tithonia diversifolia* ). Black arrows indicate the environmental variables included in the site‐specific model. *R* ^2^ values show the proportion of variance explained by these variables. Axis labels indicate the percentage of variance explained by each dbRDA axis.
**Figure S5a:** Fungal community α‐diversity (observed richness) across organic resource treatments and sites. Box plots show observed richness across four sites (Aludeka, Embu, Machanga, Sidada) and four treatments CT = control without organic inputs, FYM = farmyard manure, MS = maize ( *Zea mays* ) stover, TD = *Tithonia diversifolia* . The top panel shows combined data across all sites, while lower panels show site‐specific results. Different letters indicate significant differences between organic resource treatments (pairwise PERMANOVA, *p* < 0.05). Outliers are shown as individual points beyond the whiskers.
**Figure S5b:** Fungal community α‐diversity (evenness) across organic resource treatments and sites. Box plots show observed richness across four sites (Aludeka, Embu, Machanga, Sidada) and four treatments CT = control without organic inputs, FYM = farmyard manure, MS = maize ( *Zea mays* ) stover, TD = *Tithonia diversifolia* . The top panel shows combined data across all sites, while lower panels show site‐specific results. Different letters indicate significant differences between organic resource treatments (pairwise PERMANOVA, *p* < 0.05). Outliers are shown as individual points beyond the whiskers.
**Figure S5c:** Fungal community α‐diversity (Shannon diversity) across organic resource treatments and sites. Box plots show observed richness across four sites (Aludeka, Embu, Machanga, Sidada) and four treatments CT = control without organic inputs, FYM = farmyard manure, MS = maize ( *Zea mays* ) stover, TD = *Tithonia diversifolia* . The top panel shows combined data across all sites, while lower panels show site‐specific results. Different letters indicate significant differences between organic resource treatments (pairwise PERMANOVA, *p* < 0.05). Outliers are shown as individual points beyond the whiskers.
**Figure S5d:** Site‐specific fungal community structure by treatment. Canonical analysis of principal coordinates (CAP) constraining differences in fungal community structure by organic resource treatment at each site: (A) Sidada, (B) Aludeka, (C) Machanga, and (D) Embu. Points represent individual samples colored by treatment (red = CT control without organic input, blue = FYM farmyard manure, green = MS maize stove, purple = TD *Tithonia*). The CAP reclassification success rates providing a quantitative estimation of the degree of discrimination between organic resource treatments are shown within ellipses for each treatment group. The CAP equivalent to Pillai's trace test (with *p*‐value in brackets) indicating the overall effect size is provided at the top of each plot.
**Figure S5e:** Site‐specific fungal community structure across organic resource treatments revealed through distance‐based redundancy analysis (db‐RDA), shown for fungal communities at four sites: Aludeka, Embu, Machanga, and Sidada. Points represent individual samples colored by treatment (CT = control without organic input, FYM = farmyard manure, MS = maize stover, TD = *Tithonia diversifolia* ). Black arrows indicate the environmental variables included in the site‐specific model. *R* ^2^ values show the proportion of variance explained by these variables. Axis labels indicate the percentage of variance explained by each dbRDA axis.

## Data Availability

The raw sequencing reads of this study have been deposited in the European Nucleotide Archive (ENA) at EMBL‐EBI under accession number PRJEB103795 (https://www.ebi.ac.uk/ena/browser/view/PRJEB103795). Other data supporting the findings of this study are available within the Zenodo repository at https://doi.org/10.5281/zenodo.18891611.
