# Supplementary material for: Can Organic Amendments Override Environmental Filtering of Soil Microbiomes? Evidence From 20 Years Old Agricultural Field Trials in Contrasting Tropical Sites
Source: Glob Chang Biol. 2026 Aug 29;32(9):e71061. doi: 10.1111/gcb.71061 (PMC13525719; doi:10.1111/gcb.71061)
Supplement: Supplementary file 1 [file GCB-32-e71061-s001.docx]

# **Supplementary table S1**

Primers used for quantitative PCR (qPCR) and amplicon sequencing of prokaryotic and fungal marker genes.

| **Quantitative PCR** | | | | | | |
| --- | --- | --- | --- | --- | --- | --- |
| **Target Gene/Group** | **Primer Name** | **Sequence (5' → 3')** | **Target Region** | **Annealing Temp (°C)** | **Cycles** | **Reference** |
| Bacteria/Archaea | 515F | GTGYCAGCMGCCGCGGTAA | 16S V4 | 52 | 35 | **Parada et al., 2016** |
|  | 806R | GGACTACNVGGGTWTCTAAT |  |  |  | **Frey et al., 2016** |
| Archaeal amoA (AOA) | crenamoA19F | ATGGTCTGGCTWAGACG | amoA | 55 | 35 | **Leininger et al., 2006** |
|  | crenamoA616R | GCCATCCATCTGTATGTCCA |  |  |  | **Tourna et al., 2008** |
| Bacterial amoA (AOB) | amoA-1F | GGGGTTTCTACTGGTGGT | amoA | 55 | 35 | **Rotthauwe et al., 1997** |
|  | amoA-2R | CCCCTCKGSAAAGCCTTCTTC |  |  |  |  |
| nifH | PolF | TGCGAYCCSAARGCBGACTC | nifH | 60 | 35 | **Poly et al., 2001** |
|  | PolR | ATSGCCATCATYTCRCCGGA |  |  |  |  |
| nirK | nirK876 | ATYGGCGGVCAYGGCGA | nirK | 63, -1 per cycle  58 | 6  30 | **Henry et al., 2004** |
|  | nirK1040 | GCCTCGATCAGRTTRTGGTT |  |  |  |  |
| nirS | cd3aF | GTSAACGTSAAGGARACSGG | nirS | 63, -1 per cycle  58 | 6  30 | **Throbäck et al., 2004** |
|  | R3cd | GASTTCGGRTGSGTCTTGA |  |  |  |  |
| nosZI | nosZ1840F | CGCRACGGCAASAAGGTSMSSGT | nosZ | 65, -1 per cycle  60 | 6  30 | **Henry et al., 2006** |
|  | nosZ2090R | CAKRTGCAKSGCRTGGCAGAA |  |  |  |  |
| **Amplicon Sequencing** | | | | | | |
| Bacteria/  Archaea | 341F | CCTAYGGGDBGCWSCAG | 16S V3-V4 | 58 | 30 | **Frey et al., 2016** |
|  | 806R | GGACTACNVGGGTHTCTAAT |  |  |  |  |
| Fungi | ITS3ngs | CANCGATGAAGAACGYRG | ITS2 | 55 | 35 | **Tedersoo and Lindahl, 2016** |
|  | ITS4ngs | CCTSCSCTTANTDATATGC |  |  |  |  |

**References for table S1**

Frey, B., Rime, T., Phillips, M., Stierli, B., Hajdas, I., Widmer, F., Hartmann, M., 2016. Microbial diversity in European alpine permafrost and active layers. FEMS Microbiol. Ecol. 92, fiw018. [**https://doi.org/10.1093/femsec/fiw018**](https://doi.org/10.1093/femsec/fiw018)

Henry, S., Baudoin, E., López-Gutiérrez, J.C., Martin-Laurent, F., Brauman, A., Philippot, L., 2004. Quantification of denitrifying bacteria in soils by nirK gene targeted real-time PCR. J. Microbiol. Methods 59, 327–335. [**https://doi.org/10.1016/j.mimet.2004.07.002**](https://doi.org/10.1016/j.mimet.2004.07.002)

Henry, S., Bru, D., Stres, B., Hallet, S., Philippot, L., 2006. Quantitative detection of the *nosZ* gene, encoding nitrous oxide reductase, and comparison of the abundances of 16S rRNA, narG, nirK, and nosZ genes in soils. Appl. Environ. Microbiol. 72, 5181–5189. [**https://doi.org/10.1128/AEM.00231-06**](https://doi.org/10.1128/AEM.00231-06)

Leininger, S., Urich, T., Schloter, M., Schwark, L., Qi, J., Nicol, G. W., Prosser, J.I., Schuster, S. C., Schleper, C., 2006. Archaea predominate among ammonia-oxidizing prokaryotes in soils. Nature 442, 806–809. <https://doi.org/10.1038/nature04983>

Parada, A.E., Needham, D.M., Fuhrman, J.A., 2016. Every base matters: assessing small subunit rRNA primers for marine microbiomes with mock communities, time series and global field samples. Environ. Microbiol. 18, 1403–1414. [**https://doi.org/10.1111/1462-2920.13023**](https://doi.org/10.1111/1462-2920.13023)

Poly, F., Monrozier, L.J., Bally, R., 2001. Improvement in the RFLP procedure for studying the diversity of nifH genes in communities of nitrogen fixers in soil. Res. Microbiol. 152, 95–103. [**https://doi.org/10.1016/S0923-2508(00)01172-4**](https://doi.org/10.1016/S0923-2508(00)01172-4)

Rotthauwe, J.H., Witzel, K.P., Liesack, W., 1997. The ammonia monooxygenase structural gene amoA as a functional marker: molecular fine-scale analysis of natural ammonia-oxidizing populations. Appl. Environ. Microbiol. 63, 4704–4712. [**https://doi.org/10.1128/aem.63.12.4704-4712.1997**](https://doi.org/10.1128/aem.63.12.4704-4712.1997)

Tedersoo, L., Lindahl, B., 2016. Fungal identification biases in microbiome projects. Environ. Microbiol. Rep. 8, 774–779. [**https://doi.org/10.1111/1758-2229.12438**](https://doi.org/10.1111/1758-2229.12438)

Throbäck, I.N., Enwall, K., Jarvis, Å., Hallin, S., 2004. Reassessing PCR primers targeting nirS, nirK and nosZ genes for community surveys of denitrifying bacteria with DGGE. FEMS Microbiol. Ecol. 49, 401–417. [**https://doi.org/10.1016/j.femsec.2004.04.011**](https://doi.org/10.1016/j.femsec.2004.04.011)

Tourna, M., Freitag, T. E., Nicol, G. W., and Prosser, J. I., 2008. Growth, activity and temperature responses of ammonia-oxidizing archaea and bacteria in soil microcosms. Environ. Microbiol. 10, 1357–1364. <https://doi.org/10.1111/j.1462-2920.2007.01563.x>

Vainio, E.J., Hantula, J., 2000. Direct analysis of wood-inhabiting fungi using denaturing gradient gel electrophoresis of amplified ribosomal DNA. Mycol. Res. 104, 927–936. [**https://doi.org/10.1017/S0953756200002471**](https://doi.org/10.1017/S0953756200002471)

# **Supplementary Table S2**

Dry matter-based measurements chemical characteristics and 95% confidence intervals of organic resources applied to all sites. Measurements were available from Embu and Machanga from 2002 to 2004, and all sites from 2005 to 2007 and in 2018. (Adapted from Laub et al., 2023a Laub et al. 2023b http://creativecommons.org/licenses/by/4.0/).

| **Measured property abbreviation** | **Tithonia** | **Maize stover** | **Farmyard manure** |
| --- | --- | --- | --- |
| C (g kg^-1^)* | 345 (333 - 357) | 397 (386 - 449) | 234 (213 - 255) |
| N (g kg^-1^)* | 33.2 (28.9 - 38.2) | 7.2 (6.5 - 8) | 18.1 (15 - 21.8) |
| C/N ratio | 12.4 (10.8 - 14.1) | 58.7 (52.8 – 65.2) | 12.3 (9.9 – 15.4) |
| P (g kg^-1^)** | 2.3 (1.8 – 2.9) | 0.4 (0.3 – 0.6) | 3.1 (2.3 – 3.9) |
| K (g kg^-1^)** | 37.2 (21.2 – 65.2) | 9 (5 – 15.3) | 19.4 (7.8 – 48.6) |
| Lignin (g kg^-1^)** | 90 (62-117) | 48 (37-60) | 198 (154-242) |
| Polyphenols (g kg^-1^) | 19 (14.9 – 24.3) | 11.3 (9.5 – 13.6) | 7.8 (5.2 – 11.5) |
| Lignin/N ratio | 2.6 (1.8 -3.7) | 6.2 (4.8-8) | 6.9 (3.9 – 12.3) |
| Quality/ turnover speed*** | High / fast | Low/ fast | n.c. |
| Total N applied (kg ha^-1^ yr^-1^), -N [+N] | 323 [563] | 68 [535] | 324 [564] |
| * By dry combustion (CHN628, LECO Corporation, Michigan, USA); **Total digestion (P, K), acid detergent fiber (lignin) and Folin-Denis (polyphenols) according to Anderson and Ingram (1993); *** according to Palm et al. (2001a); values are N applied via the organic input (−N plots); values in brackets include mineral N fertilizer (+N plots) | | | |

# **Supplementary Table S3**

qPCR standard curve parameters (slope, intercept, amplification efficiency in %, and R squared) for each target gene.

|  | **16S** | **AOA** | **AOB** | **nirK*** | **nirS** | **nosZI** | **nifH** |
| --- | --- | --- | --- | --- | --- | --- | --- |
| Slope | -3.48 | -3.77 | -3.33 | -4.55 | -3.79 | -3.38 | -3.68 |
| Intercept | 9.03 | 9.43 | 8.16 | 1.24 | 1.36 | 2.69 | 6.14 |
| Efficiency | 93.68 | 84.06 | 99.58 | 65.85 | 83.45 | 97.69 | 86.81 |
| R squared | 0.99 | 0.99 | 1.00 | 0.99 | 0.99 | 1.00 | 1.00 |
| **The nirK assay had a lower amplification efficiency (66%) than the other targets (84 to 100%); nirK abundances should therefore be interpreted with caution.* | | | | | | | |

# **Supplementary Table S4**

Percentage change in microbial diversity indices and functional gene abundances under organic amendments relative to the unamended control, by site. FYM = farmyard manure, MS = maize stover, TD = Tithonia diversifolia. Values are % change of treatment means relative to control (pooled across mineral N levels and replicates).

| **Metric** | **FYM (%)** | **MS (%)** | **TD (%)** |
| --- | --- | --- | --- |
| **Aludeka** | | | |
| Bacterial richness | 43.8 | 4.4 | 33.7 |
| Bacterial diversity (H') | 8.8 | 2.0 | 11.6 |
| Fungal richness | 24.4 | 0.4 | 2.0 |
| Fungal diversity (H') | 10.8 | 4.9 | -12.3 |
| 16S rRNA | -11.4 | 14.1 | -17.0 |
| AOA (archaeal amoA) | -43.4 | -22.0 | -40.7 |
| AOB (bacterial amoA) | 92.5 | 91.0 | 198.9 |
| nifH | -52.8 | -52.7 | -59.4 |
| nirK | -2.9 | -9.5 | -16.7 |
| nirS | 90.4 | 28.8 | 28.4 |
| nosZI | 25.8 | 14.7 | 16.0 |
| **Embu** | | | |
| Bacterial richness | 60.4 | 28.8 | 11.3 |
| Bacterial diversity (H') | 4.0 | 4.0 | 2.7 |
| Fungal richness | 2.0 | 8.3 | -0.1 |
| Fungal diversity (H') | -4.7 | -1.7 | -11.7 |
| 16S rRNA | 38.7 | 128.2 | 41.5 |
| AOA (archaeal amoA) | 95.3 | 71.5 | 30.9 |
| AOB (bacterial amoA) | 217.5 | 157.0 | 98.4 |
| nifH | 46.8 | 114.7 | 40.9 |
| nirK | 24.8 | 66.0 | 19.6 |
| nirS | 31.8 | 82.3 | 22.7 |
| nosZI | 39.2 | 90.8 | 28.4 |
| **Machanga** | | | |
| Bacterial richness | 62.7 | 47.0 | 74.0 |
| Bacterial diversity (H') | 15.7 | 7.0 | 10.2 |
| Fungal richness | 34.7 | 18.4 | 47.2 |
| Fungal diversity (H') | 13.2 | 6.2 | 7.5 |
| 16S rRNA | 534.9 | 117.5 | 192.5 |
| AOA (archaeal amoA) | 835.1 | 216.9 | 416.2 |
| AOB (bacterial amoA) | 2920.4 | 148.3 | 486.1 |
| nifH | 161.2 | -15.6 | 30.8 |
| nirK | 435.6 | 62.1 | 112.4 |
| nirS | 996.7 | 48.9 | 102.6 |
| nosZI | 1192.5 | 97.8 | 191.4 |
| **Sidada** | | | |
| Bacterial richness | 77.6 | 17.3 | 36.0 |
| Bacterial diversity (H') | 17.7 | 4.4 | 3.6 |
| Fungal richness | 13.7 | 1.0 | -12.2 |
| Fungal diversity (H') | 2.7 | -1.7 | -10.7 |
| 16S rRNA | 310.0 | 39.8 | 38.3 |
| AOA (archaeal amoA) | 662.0 | 76.0 | 72.9 |
| AOB (bacterial amoA) | 1364.7 | 100.6 | 411.5 |
| nifH | 223.4 | 62.7 | 76.0 |
| nirK | 271.0 | 59.9 | 64.1 |
| nirS | 1046.5 | 62.2 | 101.9 |
| nosZI | 726.8 | 77.8 | 68.0 |

# Supplementary Table S5**.**

Summary of significant organic resource treatment effects within each site, for microbial diversity indices and N-cycling functional gene abundances. Cells give the amendment(s) differing significantly from the unamended control at that site (↑ higher, ↓ lower; ns = no significant difference), based on the pairwise comparisons shown in Figures S1a–g, S2a–c and S3a–c. FYM = farmyard manure, MS = maize stover, TD = Tithonia diversifolia. Site clay content and mean annual rainfall are given for reference.

| **Response variable** | **Aludeka** | **Embu** | **Machanga** | **Sidada** |
| --- | --- | --- | --- | --- |
|  | 5% clay, 1660 mm | 81% clay, 1175 mm | 5% clay, 795 mm | 54% clay, 1730 mm |
| **Prokaryotic α-diversity** | | | | |
| Observed richness | FYM ↑ | FYM, MS ↑ | FYM, MS, TD ↑ | FYM ↑ |
| Evenness | FYM, TD ↑ | ns | FYM ↑ | FYM ↑ |
| Shannon diversity | FYM, TD ↑ | ns | FYM, MS, TD ↑ | FYM ↑ |
| **Fungal α-diversity** | | | | |
| Observed richness | ns | ns | FYM, MS, TD ↑ | ns |
| Evenness | ns | TD ↓ | ns | TD ↓ |
| Shannon diversity | ns | TD ↓ | FYM, TD ↑ | TD ↓ |
| **N-cycling functional genes** | | | | |
| 16S rRNA | ns | MS ↑ | FYM ↑ | FYM ↑ |
| AOA (archaeal amoA) | ns | ns | ns | FYM ↑ |
| AOB (bacterial amoA) | ns | ns | FYM ↑ | ns |
| nifH | ns | MS ↑ | FYM ↑ | ns |
| nirK | ns | ns | FYM ↑ | ns |
| nirS | ns | ns | FYM ↑ | ns |
| nosZI | ns | ns | FYM ↑ | ns |

**Figure S1.** qPCR amplification curves (relative fluorescence units, RFU, versus cycle number) for the no-template blank, the plasmid standard alone (plasmid in water), and the plasmid spiked into the DNA extract of each sample, with lines showing means and shaded bands the variation across replicates.

**Figure S2.** qPCR standard curves showing quantification cycle (Cq) versus log10 standard concentration for each target gene (16S, AOA, AOB, nifH, nirK, nirS, nosZI), with points showing measured standards and lines the linear regression per target.

# **Annex I. Supplementary figures for N-cycling functional gene abundance.**

**Figure S3a.** 16 S abundances across organic resource treatments (treatment) and sites, with and without mineral nitrogen application (N). Barplots show total bacterial gene copies per gram soil across four sites (Aludeka, Embu, Machanga, Sidada) four organic resource treatments (CT = control, without organic inputs, FYM = farmyard manure, MS = maize (*Zea mays*) stover, TD = *Tithonia diversifolia*). Dark colours represent nitrogen addition (+N) and light colours represent no nitrogen addition (-N). Different letters indicate significant differences between treatments (pairwise PERMANOVA, p < 0.05).

**Figure S3b.** Ammonia-oxidizing archaea (AOA) gene abundances across organic resource treatments (treatment) and sites, with and without mineral nitrogen application (N). Barplots show total bacterial gene copies per gram soil across four sites (Aludeka, Embu, Machanga, Sidada) four organic resource treatments (CT = control, without organic inputs, FYM = farmyard manure, MS = maize (*Zea mays*) stover, TD = *Tithonia diversifolia*). Dark colours represent nitrogen addition (+N) and light colours represent no nitrogen addition (-N). Different letters indicate significant differences between treatments (pairwise PERMANOVA, p < 0.05).

**Figure S3c.** Ammonia-oxidizing bacteria (AOB) gene abundances across organic resource treatments (treatment) and sites, with and without mineral nitrogen application (N). Barplots show total bacterial gene copies per gram soil across four sites (Aludeka, Embu, Machanga, Sidada) four organic resource treatments (CT = control, without organic inputs, FYM = farmyard manure, MS = maize (*Zea mays*) stover, TD = *Tithonia diversifolia*). Dark colours represent nitrogen addition (+N) and light colours represent no nitrogen addition (-N). Different letters indicate significant differences between treatments (pairwise PERMANOVA, p < 0.05).

**Figure S3d.** Nitrogen-fixing bacteria (nifH) gene abundances across organic resource treatments (treatment) and sites, with and without mineral nitrogen application (N). Barplots show total bacterial gene copies per gram soil across four sites (Aludeka, Embu, Machanga, Sidada) four organic resource treatments (CT = control, without organic inputs, FYM = farmyard manure, MS = maize (*Zea mays*) stover, TD = *Tithonia diversifolia*). Dark colours represent nitrogen addition (+N) and light colours represent no nitrogen addition (-N). Different letters indicate significant differences between treatments (pairwise PERMANOVA, p < 0.05).

**Figure S3e.** Nitrite-reducing bacteria (nirK) gene abundances across organic resource treatments (treatment) and sites, with and without mineral nitrogen application (N). Barplots show total bacterial gene copies per gram soil across four sites (Aludeka, Embu, Machanga, Sidada) four organic resource treatments (CT = control, without organic inputs, FYM = farmyard manure, MS = maize (*Zea mays*) stover, TD = *Tithonia diversifolia*). Dark colours represent nitrogen addition (+N) and light colours represent no nitrogen addition (-N). Different letters indicate significant differences between treatments (pairwise PERMANOVA, p < 0.05).

**Figure S3f.** Nitrite-reducing bacteria (nirS) gene abundances across organic resource treatments (treatment) and sites, with and without mineral nitrogen application (N). Barplots show total bacterial gene copies per gram soil across four sites (Aludeka, Embu, Machanga, Sidada) four organic resource treatments (CT = control, without organic inputs, FYM = farmyard manure, MS = maize (*Zea mays*) stover, TD = *Tithonia diversifolia*). Dark colours represent nitrogen addition (+N) and light colours represent no nitrogen addition (-N). Different letters indicate significant differences between treatments (pairwise PERMANOVA, p < 0.05).

**Figure S3g.** Nitrous oxide-reducing bacteria (nosZI) gene abundances across organic resource treatments (treatment) and sites, with and without mineral nitrogen application (N). Barplots show total bacterial gene copies per gram soil across four sites (Aludeka, Embu, Machanga, Sidada) four organic resource treatments (CT = control, without organic inputs, FYM = farmyard manure, MS = maize (*Zea mays*) stover, TD = *Tithonia diversifolia*). Dark colours represent nitrogen addition (+N) and light colours represent no nitrogen addition (-N). Different letters indicate significant differences between treatments (pairwise PERMANOVA, p < 0.05).

# **Annex II. Supplementary figures of prokaryotic α- and β-diversity.**

**Figure S4a.** Prokaryotic community α-diversity (observed richness) across organic resource treatments and sites. Box plots show observed richness across four sites (Aludeka, Embu, Machanga, Sidada) and four treatments CT = control without organic inputs, FYM = farmyard manure, MS = maize (*Zea mays*) stover, TD = *Tithonia diversifolia*). The top panel shows combined data across all sites, while lower panels show site-specific results. Different letters indicate significant differences between organic resource treatments (pairwise PERMANOVA, p < 0.05). Outliers are shown as individual points beyond the whiskers.

**Figure S4b.** Prokaryotic community α-diversity (evenness) across organic resource treatments and sites. Box plots show observed richness across four sites (Aludeka, Embu, Machanga, Sidada) and four treatments CT = control without organic inputs, FYM = farmyard manure, MS = maize (*Zea mays*) stover, TD = *Tithonia diversifolia*). The top panel shows combined data across all sites, while lower panels show site-specific results. Different letters indicate significant differences between organic resource treatments (pairwise PERMANOVA, p < 0.05). Outliers are shown as individual points beyond the whiskers.

**Figure S4c.** Prokaryotic community α-diversity (Shannon diversity) across organic resource treatments and sites. Box plots show observed richness across four sites (Aludeka, Embu, Machanga, Sidada) and four treatments CT = control without organic inputs, FYM = farmyard manure, MS = maize (*Zea mays*) stover, TD = *Tithonia diversifolia*). The top panel shows combined data across all sites, while lower panels show site-specific results. Different letters indicate significant differences between organic resource treatments (pairwise PERMANOVA, p < 0.05). Outliers are shown as individual points beyond the whiskers.

**Figure S4d.** Site-specific prokaryotic community structure by organic resource treatment. Canonical analysis of principal coordinates (CAP) constraining differences in prokaryotic community structure by treatment at each site: (A) Sidada, (B) Aludeka, (C) Machanga, and (D) Embu. Points represent individual samples coloured by organic treatment (red = CT control without organic input, blue = FYM farmyard manure, green = MS maize stove, purple = TD *Tithonia*). The CAP reclassification success rates providing a quantitative estimation of the degree of discrimination between treatments are shown within ellipses for each treatment group. The CAP equivalent to Pillai's trace test (with *p*-value in brackets) indicating the overall effect size is provided at the top of each plot.

**Figure S4e.** Site-specific prokaryotic community structure across organic resource treatments revealed through distance-based redundancy analysis (db-RDA), shown for prokaryotic communities at four sites: Aludeka, Embu, Machanga, and Sidada. Points represent individual samples coloured by treatment (CT = control, FYM = farmyard manure, MS = maize stover, TD = *Tithonia diversifolia*). Black arrows indicate the environmental variables included in the site-specific model. R² values show the proportion of variance explained by these variables. Axis labels indicate the percentage of variance explained by each dbRDA axis.

# **Annex III. Supplementary figures of fungal α- and β-diversity diversity**

**Figure S5a.** Fungal community α-diversity (observed richness) across organic resource treatments and sites. Box plots show observed richness across four sites (Aludeka, Embu, Machanga, Sidada) and four treatments CT = control without organic inputs, FYM = farmyard manure, MS = maize (*Zea mays*) stover, TD = *Tithonia diversifolia*). The top panel shows combined data across all sites, while lower panels show site-specific results. Different letters indicate significant differences between organic resource treatments (pairwise PERMANOVA, p < 0.05). Outliers are shown as individual points beyond the whiskers.

**Figure S5b.** Fungal community α-diversity (evenness) across organic resource treatments and sites. Box plots show observed richness across four sites (Aludeka, Embu, Machanga, Sidada) and four treatments CT = control without organic inputs, FYM = farmyard manure, MS = maize (*Zea mays*) stover, TD = *Tithonia diversifolia*). The top panel shows combined data across all sites, while lower panels show site-specific results. Different letters indicate significant differences between organic resource treatments (pairwise PERMANOVA, p < 0.05). Outliers are shown as individual points beyond the whiskers.

**Figure S5c.** Fungal community α-diversity (Shannon diversity) across organic resource treatments and sites. Box plots show observed richness across four sites (Aludeka, Embu, Machanga, Sidada) and four treatments CT = control without organic inputs, FYM = farmyard manure, MS = maize (*Zea mays*) stover, TD = *Tithonia diversifolia*). The top panel shows combined data across all sites, while lower panels show site-specific results. Different letters indicate significant differences between organic resource treatments (pairwise PERMANOVA, p < 0.05). Outliers are shown as individual points beyond the whiskers.

**Figure S5d.** Site-specific fungal community structure by treatment. Canonical analysis of principal coordinates (CAP) constraining differences in fungal community structure by organic resource treatment at each site: (A) Sidada, (B) Aludeka, (C) Machanga, and (D) Embu. Points represent individual samples coloured by treatment (red = CT control without organic input, blue = FYM farmyard manure, green = MS maize stove, purple = TD *Tithonia*). The CAP reclassification success rates providing a quantitative estimation of the degree of discrimination between organic resource treatments are shown within ellipses for each treatment group. The CAP equivalent to Pillai's trace test (with *p*-value in brackets) indicating the overall effect size is provided at the top of each plot.

**Figure S5e.** Site-specific fungal community structure across organic resource treatments revealed through distance-based redundancy analysis (db-RDA), shown for fungal communities at four sites: Aludeka, Embu, Machanga, and Sidada. Points represent individual samples coloured by treatment (CT = control without organic input, FYM = farmyard manure, MS = maize stover, TD = *Tithonia diversifolia*). Black arrows indicate the environmental variables included in the site-specific model. R² values show the proportion of variance explained by these variables. Axis labels indicate the percentage of variance explained by each dbRDA axis.
